# Hallmarks and detection techniques of cellular senescence and cellular ageing in immune cells

**DOI:** 10.1111/acel.13316

**Published:** 2021-02-01

**Authors:** Dingxi Zhou, Mariana Borsa, Anna Katharina Simon

**Affiliations:** ^1^ The Kennedy Institute of Rheumatology University of Oxford Oxford UK

**Keywords:** ageing markers, immunosenescence, methods

## Abstract

The ageing of the global population brings about unprecedented challenges. Chronic age‐related diseases in an increasing number of people represent an enormous burden for health and social care. The immune system deteriorates during ageing and contributes to many of these age‐associated diseases due to its pivotal role in pathogen clearance, tissue homeostasis and maintenance. Moreover, in order to develop treatments for COVID‐19, we urgently need to acquire more knowledge about the aged immune system, as older adults are disproportionally and more severely affected. Changes with age lead to impaired responses to infections, malignancies and vaccination, and are accompanied by chronic, low‐degree inflammation, which together is termed immunosenescence. However, the molecular and cellular mechanisms that underlie immunosenescence, termed immune cell senescence, are mostly unknown. Cellular senescence, characterised by an irreversible cell cycle arrest, is thought to be the cause of tissue and organismal ageing. Thus, better understanding of cellular senescence in immune populations at single‐cell level may provide us with insight into how immune cell senescence develops over the life time of an individual. In this review, we will briefly introduce the phenotypic characterisation of aged innate and adaptive immune cells, which also contributes to overall immunosenescence, including subsets and function. Next, we will focus on the different hallmarks of cellular senescence and cellular ageing, and the detection techniques most suitable for immune cells. Applying these techniques will deepen our understanding of immune cell senescence and to discover potential druggable pathways, which can be modulated to reverse immune ageing.

## Graphical abstract

This review comprehensively summarises hallmarks of cellular senescence and cellular ageing, and for the first time, the detection techniques most suitable for immune cells. The application of these techniques will enable us to gain a deeper understanding of immune cell senescence, and potentially, an avenue to reverse immune ageing.

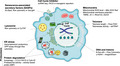

## 1 INTRODUCTION

Organismal ageing is caused by the progressive deterioration of most cells, tissues or organs of the body. The immune system is not an exception. Its dysregulation and deterioration, so‐called ‘immunosenescence’, predispose older adults to a diminished response to infections with novel pathogens, to autoimmunity, as well as to chronic non‐immune disorders including cardiovascular and neurodegenerative diseases, cancers and type‐2 diabetes (Fulop et al., 2016; Simon et al., 2015). Immunosenescence is a combination of immune system‐wide changes over the life time of an individual, which are much better studied (examples can be found in the next paragraph on T and B cells), and cellular changes in individual immune cells, which we will term immune cell senescence. To be able to establish to which degree immune cell senescence contributes to immunosenescence, it will be important to first establish which immune cells undergo cellular senescence with age, as it is much less studied. This review will focus on techniques to detect the cellular changes leading to immune cell senescence.

A recent landmark study by Alpert et al. (2019) which has used ‘multi‐omics’ technologies to uncover the cell subsets linked with age, provides longitudinal metrics of immune age. In addition, several recent reviews on the topic of immune system‐wide changes give extensive overviews of surface markers and cell populations that emerge during ageing (Alpert et al., 2019; Drew et al., 2018; Frasca, 2018; Judge et al., 2020; Xu & Larbi, 2017; Yarbro et al., 2020). Thus, here we will only briefly summarise some of the most important immune system‐wide changes and focus on the available strategies that can be used to detect them.

T and B lymphocytes are major players of the adaptive immune system (Lanier, 2013). Their diverse antigen‐recognition repertoire enables naïve T and B cells to respond specifically to a variety of foreign antigens. Their antigen‐experienced memory population provides long‐lasting protection by responding to previously encountered antigens in a more rapid and robust manner. However, during ageing, the production of naïve lymphocytes declines, shrinking the repertoire of antigen‐specific cells able to generate new memory cells (Salam et al., 2013). This compromises the ability of an aged immune system to fight against new pathogens and establish memory responses to these *de novo* antigens. Meanwhile, antigen‐experienced cells accumulate and undergo oligoclonal expansion in the aged individuals, reflecting lymphopenia‐driven homeostatic proliferation, the adaptive response from a reduced naïve pool and the effect of past and persistent infections. Some subsets of lymphocytes, characterised by reduced antigen‐receptor signalling and innate‐like phenotypes, are significantly increased in frequency during ageing. Due to their altered function and low proliferation rates, many of these highly inflammatory cells were the first to be termed senescent, including effector memory T cells re‐expressing CD45RA (T_EMRA_), and late/exhausted memory B cells (LM B cells) (Callender et al., 2018; Colonna‐Romano et al., 2009; Di Mitri et al., 2011; Frasca et al., 2017; Lanna et al., 2014). However, whether they are truly ‘senescent’ is still putative, since it has been reported that they, or their subpopulation, are able to proliferate under specific conditions (Di Mitri, 2011; Hao et al., 2011; Verma et al., 2017). Age‐associated changes also correlate with the expression of certain surface molecules, which can be detected using antibody staining via flow cytometry. One example is the increased expression of CD57 in T cells (and natural killer cells, which belong to the innate immune system) during ageing, which has been linked with senescent‐like phenotypes (Alpert et al., 2019; Brenchley et al., 2003; Lopez‐Verges et al., 2010). Downregulation of CD27 and CD28, and upregulation of KLRG1 are also linked with functionally deficient T cells (Henson & Akbar, 2009; Plunkett et al., 2007). In Table 1, we summarise these T and B cells that accumulate with age and their surface markers.

**TABLE 1:** Subtypes of T and B cells display senescent‐like phenotypes and accumulate during ageing

	Senescent‐like population	Surface marker	Tissue	Species (Human/Murine)	Percentage of total T or B cells		Characteristics related to cellular senescence
					Young	Old	
T cell	Senescent‐like CD8^+^ T cells (Henson, 2009)	CD8^+^ CD27^‐^ CD28^‐^	PBMC	Human	0.5%	50%	↓telomere length (Plunkett, 2007); ↓telomerase activity; ↓proliferation capacity
	Terminally differentiated CD8^+^ T cell (T_EMRA_) (Le Page et al., 2018)	CD8^+^ CD45RA^+^ CCR7^‐^	PBMC	Human	3%	14%	↓proliferation capacity (Callender et al., 2018) ↑pro‐inflammatory cytokine
	CD57^+^ KLRG1^+^ CD8^+^ T cells (Onyema, 2012)	CD8^+^ CD57^+^ KLRG1^+^	PBMC	Human	7.9%	32.2%	↓proliferation capacity (Brenchley et al., 2003)
	Virtual memory CD8^+^ T cells (Tvm)	CD45RA^+^ PanKIR^+^ and/or NKG2A^+^ (Quinn, 2018)	PBMC	Human	4%	12%	↓proliferative capacity; ↑γH2AX
		CD44^hi^ CD49d^lo^ (Quinn, 2018)	Spleen	Murine	10%	30%	
B cell	Age‐associated B cells	CD21/35^‐^ CD23^‐^ CD43^‐^ CD93^‐^ (Hao et al., 2011)	Spleen	Murine, female	3%	12%	↓proliferation capacity ↑pro‐inflammatory cytokine
	Age‐associated B cell‐like cells	CD11c^+^ CD11b^+^ CD21^‐^ (Rubtsov, 2011)	Spleen	Murine, female	0.25%	6.64%	NA

The deleterious effect of ageing on the adaptive immune system has been extensively investigated (Pawelec et al., 1999). However, there is accumulating evidence suggesting that innate immune systems are also affected, contributing to the emergence of age‐related phenotypes in the elderly (Ray & Yung, 2018). Monocytes/macrophages, dendritic cells and natural killer cells have all been reported to undergo changes in number and phenotype during ageing (Almeida‐Oliveira et al., 2011; Bhushan et al., 2002; Hearps et al., 2012; Nyugen et al., 2010; Seidler et al., 2010). The age‐related myeloid bias during haematopoiesis can explain the increased numbers of macrophages and dendritic cells to a certain extent. Some explanations for the myeloid bias have been put forward, including: (1) a cell‐intrinsic issue with the aged hematopoietic stem cell (Pang et al., 2011), (2) the aged microenvironment (Ergen et al., 2012), and (3) increased number of senescent cells that leads to an increase in macrophages and NK cells for their removal (Hall et al., 2016; Sagiv et al., 2016).

Many of their functions are also affected, and these are more likely to be due to immune cell senescence. For example, monocytes and neutrophils from aged humans (or mice) display decreased phagocytosis and ROS production, which impairs their antimicrobial capacity (Butcher et a., 2001; Hearps et al., 2012; McLachlan et al., 1995; Wenisch et al., 2000). The antigen‐presenting ability of dendritic cells relies on their ability to phagocytose, which declines with age and may further affect the establishment of adaptive immunity (Agrawal et al., 2007). Another feature of an aged innate immune cell is the dysregulated cytokine/chemokine secretion, mainly studied in macrophages (and recently in T cells), which contributes to fuel the pro‐inflammatory environment observed during ageing, termed ‘inflamm‐aging’ (Desdin‐Mico et al., 2020; Franceschi et al., 2017; Hearps et al., 2012; Qian et al., 2012).

Inflamm‐aging refers to chronic, low‐grade inflammation and is possibly caused by prolonged infection, dysregulated metabolism and accumulation of senescent cells (Franceschi et al., 2000, 2018). Some inflammatory molecules in blood, including pro‐inflammatory cytokines, chemokines and acute‐phase proteins, are widely used as readouts to assess inflamm‐aging (Franceschi et al., 2017). From an evolutionary perspective, inflammation plays an important role in the short‐term clearance of pathogens in early life (Franceschi et al., 2000). However, the chronic inflammation with age results in accelerated systemic ageing, leading to negative physiological outcomes that are not limited to immunity, which reinforces the pleiotropy theory of ageing.

Although many of the mentioned alterations have been associated with immune ageing, it remains largely unknown whether they are signs of maladaptation, or the outcome of an adaptation to compensate for a dysregulated immune system. To address this, a detailed profiling of cellular and molecular changes over the lifetime of an individual is essential. Do all changes happen to each cell at once, or just to some and in subsequent steps and does that correlate with function? The good news is that the emerging field of geroscience may be of considerable help. Geroscience assumes that mechanisms underlying ageing and age‐related diseases are largely overlapping and shared across cell types (Franceschi et al., 2018). Nowadays, several interconnected ‘pillars’ promoting ageing have been identified, including mitochondrial health, DNA damage, telomere shortening, chromosome reorganisation, cell cycle arrest, excessive cytokine release, proteostasis and lysosomal health (Kennedy et al., 2014). Applying the wide range of methods that have been developed and tested in multiple tissues and organisms to the immune system will provide important insights into the molecular basis of immunosenescence.

The current COVID‐19 pandemic affects mostly the elderly and as such is closely linked to immunosenescence. A case report profiled the kinetics of immune responses in response to SARS‐CoV‐2 infection. Both innate and adaptive immunological changes were detected before the full resolution of symptoms (Thevarajan et al., 2020). The remodelling of the immune systems during ageing may explain why older adults are more at risk to develop severe COVID‐19. Two hallmarks of ageing, which are, shorter telomere length and aberrant mitochondria function, have been hypothesised to indicate a worse prognosis in COVID‐19 (Aviv et al., 2020; Kloc et al., 2020). Systematically screening the hallmarks of both immune cell senescence and immune system‐wide changes may unravel their relationship with the progression of the disease. Vaccines against SARS‐CoV‐2 are commonly considered as the best way of protecting the elderly and putting the pandemic to an end (Lurie et al., 2020; Zhang et al., 2020). However, the successful vaccination in senior people is a big challenge, since vaccines are much less immunogenic and effective in this cohort (Weinberger et al., 2018). Therefore, understanding the molecular mechanisms that underpin immune cell senescence may help to boost the effectiveness of SARS‐CoV‐2 vaccines and protect the vulnerable older adults. Furthermore, the predisposition of aged people to inflamm‐aging may lead to immune pathology. In severe cases, patients suffer from excessive pro‐inflammatory cytokines, the so‐called ‘cytokine storm’, possibly promoted by inflamm‐aging (Meftahi et al., 2020). Indeed, dexamethasone, an anti‐inflammatory glucocorticoid, reduces the 28‐day mortality rate in a randomised clinical trial (Group et al., 2020). We believe that understanding the cellular changes that occur with age in immune cells, which may lead to aberrant responses, including inflamm‐aging, can help to identify drug targets for COVID‐19 treatment (Channappanavar & Perlman, 2020).

**FIGURE 1:**
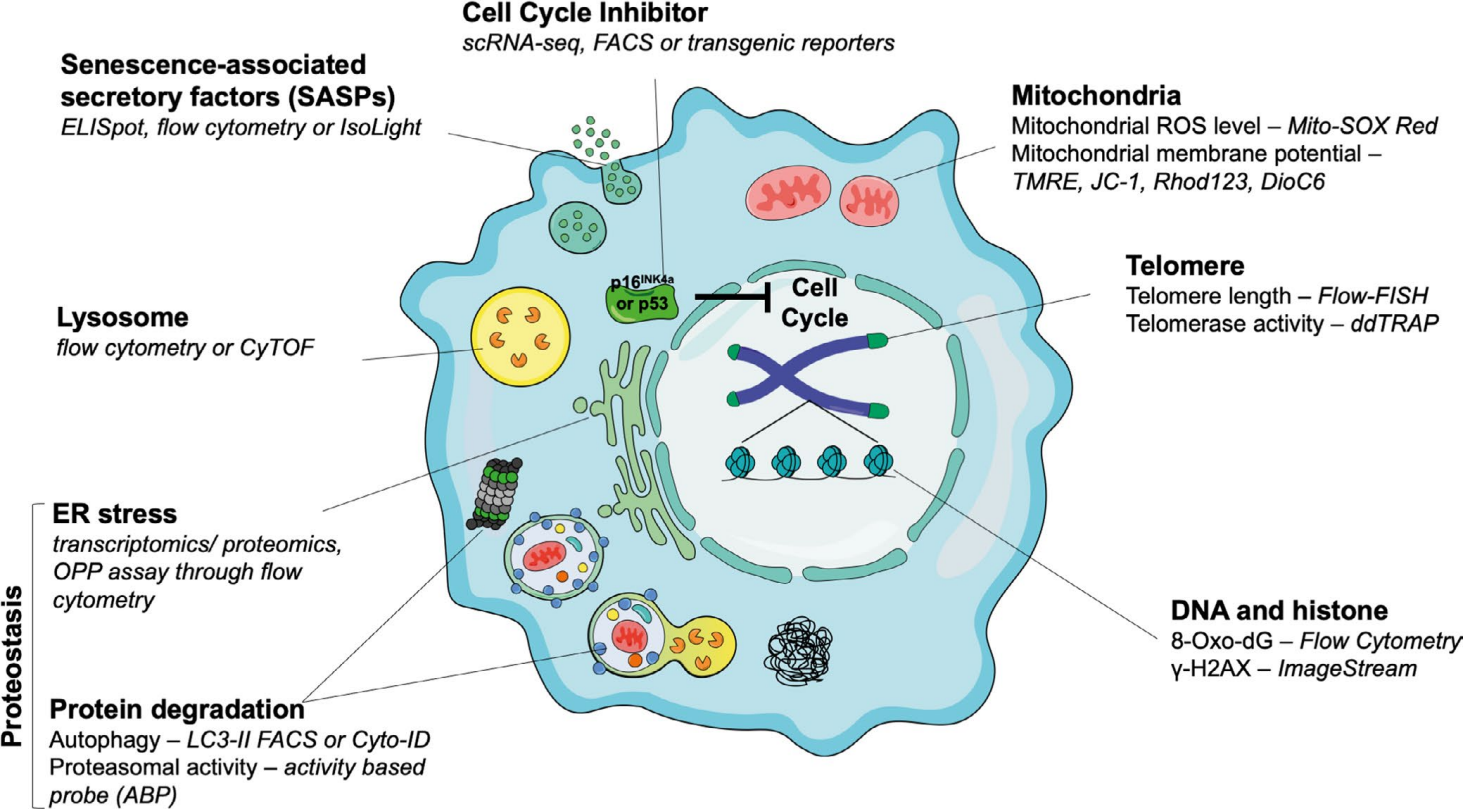
Senescence biomarkers and detection techniques. Senescent cells display excessive oxidative stress in mitochondria, which further induces DNA and protein damage and leads to disrupted proteostasis and cell cycle arrest. The lysosomal accumulation of lipofuscin and SA‐β‐Gal serves as common hallmarks for the identification of senescent cells. The dramatic change in the pro‐inflammatory secretome of senescent cells, termed senescent‐associated secretory phenotype, might contribute to inflamm‐aging, the chronic, low‐degree inflammation during ageing

In this review, we will compile the most widely used and accepted age‐related cellular changes, and highlight the most recent techniques that can be applied to different immune cell types in a high‐throughput manner (overviewed in Figure 1 and summarised in Table 2). These techniques, particularly the use of flow cytometry and omics at single‐cell resolution, will help to correlate immune cell senescence with immune dysfunction. Furthermore, application of these techniques will potentially help to unveil druggable targets, and help to design drugs to rejuvenate aged immune cells and prevent age‐related diseases. Because certain age‐associated cell changes are not necessarily characteristics of senescence, and rather are hallmarks of cellular ageing, the next chapter will be divided into hallmarks of cellular senescence and hallmarks of cellular ageing (Gorgoulis et al., 2019; Lopez‐Otin et al., 2013). The final part of this review aims to give our readers a perspective of the future directions of ageing research.

**TABLE 2:** The detection techniques of senescent biomarkers and their applications in immune cells

Category	Phenotype during ageing	Detection techniques	Phenotype in aged immune cells	References
Mitochondrial damage and oxidative stress	cellular ROS ↑	H_2_‐DCFDA or CellROX staining for flow cytometry	Increase in bone marrow‐derived and tissue‐resident macrophages	Sebastian et al. (2009) Vida et al. (2017)
	mitochondrial ROS ↑	Mito‐SOX Red staining for flow cytometry	Increase in naïve and central memory CD8^+^ T and antibody‐secreting cells	Sanderson and Simon (2017); Kurupati et al. (2019)
	mitochondrial membrane potential ↓	TMRE, JC−1, Rhodamine 123 or DioC6 staining for flow cytometry	No change in CD8^+^ T nor antibody‐secreting cells	Sanderson and Simon (2017); Kurupati et al. (2019)
p16^INK4a^ (similar to p53)	↑	Single‐cell RNA sequence	Increase in human peripheral T cells and mouse B cells	Liu et al. (2011) Liu et al. (2009)
		Antibody staining for flow cytometry or transgenic reporter mouse line	Minor increase in p16^INK4a+^ T and B cells	Liu et al. (2019)
Senescence‐associated secretory phenotype	↑	qPCR single‐cell RNA sequence	Increase in mouse senescent‐associated T cells, human T_EMRA_ cells, late/ exhausted memory B cells, mouse bone marrow myeloid cells and neutrophils	Fukushima et al. (2018) Callender et al. (2018) Frasca et al. (2017) Farr et al. (2016) Uhl et al. (2016)
		ELISpot	IL−6, TNF‐a, MMPs	
		Luminex IsoLight	NA	NA
Senescence‐associated beta‐galactosidase	**↑**	C_12_FDG or its analogue for flow cytometry or CyTOF	Increase in memory‐phenotype (CD44^high^) T cells	Liu et al. (2019)
Lipofuscin	↑	biotin‐conjugated GL13 staining for flow cytometry	Increase in peritoneal macrophages, microglia, and T cells	Vida et al. (2017) Kushwaha et al. (2018)
DNA damage	8‐Oxoguanine↑	flow cytometry	NA	Sampson et al. (2006)
	γH2AX↑	ImageStream	Increase in virtual memory T cells, human HSCs and progenitor cells	Quinn et al. (2018) Durdik et al. (2015) Phadwal et al. (2012)
Telomere	average telomere length↓	Flow‐FISH	Shortened in human peripheral T cells	Sanderson and Simon (2017) Fali et al. (2019)
	telomerase activity↓	droplet digital TRAP (ddTRAP)	Declines in resting T and B cells	Lin et al. (2015)
Ubiquitin‐proteasome system	proteasome activity↓	activity‐based probe (ABP)	Decrease in lymphocytes and T cells	Carrard et al. (2003) Ponnappan et al. (1999) Arata et al. (2019)
Autophagy	macroautophagy flux↓	LC3‐II flow cytometry	Decrease in macrophages, T and B cells	Puleston et al. (2014) Stranks et al. (2015) Bharath et al. (2020) Zhang et al. (2019)
	Chaperon‐mediated autophagy↓	KFERQ‐tagged protein reporters; Co‐stain with HSC70 and LAMP2A	Decrease in T cells	Valdor et al. (2014)
ER stress	BiP ↓ UPR ↑ Translation ↓	Western Blot or qPCR for BiP Flow cytometry or in vivo imaging for protein translation	Decrease of BiP expression and protein translation in several murine tissues	Naidoo et al. (2008) Zhang et al. (2019) Signer et al. (2014) Liu, Xu et al (2012)

## 2 HALLMARKS OF CELLULAR SENESCENCE

### 2.1 Cell Cycle

Common inducers of senescence include telomere erosion, DNA damage, oxidative stress, oncogene activation and chronic mitogen signalling. The downstream signalling cascades induced by these stressors ultimately converge to the p53/p21^CIP^ and/or the p16^INK4a^/pRB pathways, which directly act on cyclin‐dependent kinases to inhibit the cell cycle (Bruce et al., 2000). Both pathways contribute to the initial growth arrest during cellular senescence (Prieur et al., 2011). However, studies show that the type of stress signal determines which pathway takes the leading role. For instance, DNA damage upregulates p21^CIP^ levels in a p53‐dependent manner (Mlynarczyk & Fåhraeus, 2014), while p38‐MAPK‐mediated increase in mitochondrial ROS levels stimulates p16^INK4a^ expression (Luo et al., 2011).

#### 2.1.1 p16^Ink4a^

Quantitative polymerase chain reaction (qPCR) has long been the benchmark technique for measuring mRNA levels. Some studies show an increase of *p16^INK4a^* mRNA in human peripheral T cells (Liu et al., 2009), as well as mouse B cells during ageing (Liu et al., 2011). Due to the heterogeneity of senescence within the same cell population, it is necessary not only to measure the average change of *p16^INK4a^* mRNA level over many cells, but at single‐cell resolution. At present, single‐cell RNA sequencing (scRNA‐seq) allows us to identify *p16^INK4a^*‐expressing cells in a high‐throughput manner, while also revealing other differentially expressed genes (Enge et al., 2017). Applying the technique to immune cells may help to identify whether they are senescent. Transgenic p16^INK4a^‐reporter mice also shed some light on the issue, and several mouse lines generated with different reporter‐constructs are available (Demaria et al., 2014; Liu et al., 2019). A recent article demonstrates that less than 1% of T or B cells (among their total respective peripheral populations) are p16^INK4a^ positive in aged mice. This is a much lower frequency than the one found in other cell populations, such as cartilage and fat progenitor cells, among which around 6% of the cells express p16^INK4a^. Thus, in comparison with other tissues, it remains to be elucidated whether p16^INK4a^ is a reliable biomarker for senescence in immune cells by looking at other cellular changes and correlating them with immune function (Liu et al., 2019). One explanation is that the *p16^INK4a^* promoter activation does not reflect mRNA abundance. The senescent lymphocytes accumulate high levels of the *p16^INK4a^* transcript with marked stability, but due to technical difficulties p16^INK4a^ protein levels are not easy to detect with this reporter line (Liu et al., 2019).

To measure p16^INK4a^ protein expression, flow cytometry or its multiparameter derivative CyTOF (time‐of‐flight mass cytometry), which both rely highly on the specificity of the antibodies, are alternatives (Cheung & Utz, 2011). CyTOF currently can detect more than 50 features in a single cell simultaneously (Olsen et al., 2019). The high‐dimensional data generated enable more detailed characterisation of p16^INK4a+^ immune populations and identification of surface markers and ageing biomarkers correlating it with p16 expression. One hurdle is that p16^INK4a^ antibodies have not yet been validated for either method. However, other methods independent of highly specific antibodies, such as single‐cell mass spectrometry‐based proteomics, are emerging, which will help to answer whether and how p16^INK4a^ expression contributes to immune cell senescence (Dou et al., 2019).

#### 2.1.2 p53‐p21^CIP^ signalling pathway

*p53* is a tumour suppressor gene, which is frequently mutated in human cancer (Yue et al., 2017). Upon DNA damage responses, p53 protein undergoes post‐translational modification and induces cell cycle arrest and/or apoptosis through its transcription factor activity. Thus, it is crucial to the maintenance of genome stability. Similar to p16^INK4a^, p53 also participates in cellular senescence. One of its downstream genes, *p21^CIP^*, is a cyclin‐dependent kinase inhibitor, which halts the progress of the cell cycle through interacting with multiple types of cyclin‐dependent kinases (He et al., 2005). The activation of p53‐p21^CIP^ pathway has been widely observed in senescent cells, though only at the early stage, which suggest to its role in induction of cellular senescence (Stein et al., 1999). The p53 pathway has complex roles in ageing, depending on the persistence of the stimuli, which activate p53 expression (Liu & Sharpless, 2009). Transient stimuli‐triggered p53 activity contributes to genome stability through temporarily pausing cell cycle for DNA repair. However, continuous p53 activation caused by prolonged stress leads to senescence or cell death (Liu & Sharpless, 2009). Aged splenocytes and thymocytes display elevated p53 levels, suggesting the accumulation of DNA damage (Kapasi & Singhal, 1999). Different p53 isoforms can have a different impact on ageing. The age‐associated human peripheral CD8^+^ T cells (CD28^‐^ CD57^+^) show decreased Δ133p53 and increased p53β levels (Mondal et al., 2013). Reconstituting Δ133p53 expression recovers the proliferation of these age‐associated T cells and rescues their senescent phenotypes, suggesting a potential therapeutic strategy for treating immune cell senescence.

The methods to detect p16^INK4a^ and p53 are similar. Note that p53 activity is determined by its various post‐translational modifications (PTM) and isoforms (Liu et al., 2019). A systematic analysis with PTM‐ and isoform‐specific antibodies will fully uncover p53’s role in immune ageing.

### 2.2 Telomere attrition

Telomeres are special structures at the end of linear chromosomes, which, in mammalian cells, consist of typical telomeric DNA repeats (TTAGGG) and associated proteins (Shay & Wright, 2019). The protein complex prevents telomeres from being recognised as damaged DNA and represses the DNA damage response. During DNA replication, the replicate machinery cannot fully copy the telomeric DNA, leading to the shortening of telomere each time, termed ‘end‐replication problem’ (Shay & Wright, 2019). Once the telomeric DNA shortens below a threshold, it may cause DNA breaks and replicative senescence (d'Adda di Fagagna, 2003). Thus, telomere shortening, which is a cause of telomere dysfunction, can be used as a readout to detect cellular senescence (Bernadotte et al., 2016).

In the immune system, the average telomere length decreases both in lymphocytes and granulocytes with ageing, with lymphocytes experiencing more pronounced telomere shortening (Aubert et al., 2012). The telomere length also varies between different subtypes of human CD8^+^ T lymphocytes. Naïve populations usually display longer telomeres than the highly differentiated ones, leading to higher proliferative potential (Sanderson & Simon, 2017). Thus, both telomere attrition and population shift from naïve to memory and effector cells lead to the overall shortening of telomeres in T cells (Fali et al., 2019). Flow‐FISH (fluorescence in situ hybridisation) has been widely used to measure average telomere length in hematopoietic lineages (Baerlocher et al., 2006). It combines flow cytometry with the hybridisation of telomeric PNA probes to cells in suspension. Telomere length by Flow‐FISH correlates with mitochondrial stress in T cells (Sanderson & Simon, 2017) and lymphocyte function, predicting immune responses to vaccination in the elderly (Najarro et al., 2015).

Telomerase is a reverse transcriptase that maintains telomere length (Pfeiffer & Lingner, 2013). Due to their high telomerase activity, immortal cell lines, germline cells, stem cells or cells at early stage of fetal development may escape replicative senescence caused by telomere dysfunction (Liu et al., 2007; Montalto et al., 1999). While in resting lymphocytes, telomerase activity is usually low, mitogen stimulation can transiently upregulate telomerase activity (Hiyama et al., 1995). One study showed that with age telomerase activity declines in resting T and B cells (Lin et al., 2015). To measure telomerase enzyme activity at single‐cell level, traditional telomere repeat amplification protocol (TRAP) was adapted to a digital format, called ‘droplet digital TRAP’ (ddTRAP) (Ludlow et al., 2014). This method allows the quantification of telomerase‐extended products in a single cell with high accuracy and reproducibility. Several studies managed to monitor telomerase activity in single T cells with ddTRAP (Huang et al., 2017; Tedone et al., 2019).

### 2.3 Senescence‐associated secretory phenotype

The senescence‐associated secretory phenotype is another typical feature of senescent cells. Senescent cells secrete a spectrum of soluble factors including pro‐inflammatory cytokines, chemokines, growth factors and proteases. In a recent article, Nathan et al. introduces the ‘SASP atlas’, which profiles SASP factors in human lung fibroblasts and renal cortical epithelial cells (Basisty et al., 2020). In young individuals, the SASP contributes to tissue homeostasis by facilitating myofibroblast differentiation and wound healing (Demaria et al., 2014). However, as senescent cells accumulate with age, chronic SASP promotes degenerative changes in neighbouring cells and age‐related pathologies, and possibly inflamm‐ageing. This is supported by studies using senolytics that specifically eliminate senescent cells through pharmacological or genetic methods (definitive evidence revealing that senescent cells accelerate age‐related phenotypes systematically) (Baker et al., 2011; Zhu et al., 2015). Therefore, inhibiting SASP production may become a target of anti‐ageing therapies (Laberge et al., 2015).

SASP factors can potentially create a pro‐inflammatory environment and recruit immune cells (Prata et al., 2018). The immune cells may further produce more pro‐inflammatory cytokines and aggravate age‐related pathologies. Some age‐associated adaptive immune populations are able to secrete factors belonging to SASP. The terminally differentiated T_EMRA_ cells in humans, and the T_EMRA_‐like virtual memory CD8^+^ T cells in mice, which accumulate during ageing, exhibit a uniquely high inflammatory secretory profile characteristic of SASP, which is regulated by p38‐MAPK signalling pathway (Callender et al., 2018; Quinn et al., 2018). However, it is still elusive whether increased SASP serves as a homeostatic, adaptive response to the age‐associated decline in antigen‐specific immunity. The function of SASP^+^ immune cells needs to be carefully determined in vivo.

A large proportion of SASP factors may originate from the innate immune system. Aged CD14^+^ myeloid cells isolated from mouse bone marrow show increased expression of 36 established SASP‐related mRNA (Farr et al., 2016). Similarly, synthesis of pro‐inflammatory (IL‐1α, IL‐1β, IL‐6, TNFα and IFNγ) cytokines was significantly increased in aged neutrophils (Uhl et al., 2016) and surprisingly T cells contribute to age‐related pro‐inflammatory cytokine production (Desdin‐Mico et al., 2020). Although the secretome of aged immune cells overlaps with SASP, whether and how it is associated with cellular senescence still needs to be ascertained. Detailed profiling of SASP components, other hallmarks of senescence as described herein and the upstream signalling inducing such phenotype will solve this question.

To measure cytokine secretion at the single‐cell level, traditional methods involve enzyme‐linked immune absorbent spot (ELISpot) and flow cytometry‐based assays. To detect all relevant cytokines simultaneously, several techniques offer a solution (Xu et al., 2019). Cell culture supernatants containing SASPs are added to a mixture of beads (Luminex) pre‐coated with analyte‐specific capture antibodies, followed by detection with fluorescent dyes and flow‐based analysis. Though enabling multi‐analyte profiling, the method is not easily applicable to single cells. However, another technique, called IsoLight, combines sandwich ELISA and fluorescence signal detection (Liu et al., 2020) and is based on a single‐cell, highly multiplexed chip consisting of an antibody barcode array. It enables the quantification of 40+ key secreted proteins from up to ~10,000 live single cells. For surface staining, antibodies that do not affect intracellular signalling pathways are required to avoid changes in the cell secretome. Such platforms can further be adapted to include common SASP factors for ageing biomarker development.

### 2.4 Senescence‐associated beta‐galactosidase

Senescence‐associated beta‐galactosidase (SA‐β‐gal) activity, together with p16^INK4a^ expression, serves as the most widely used biomarker for detecting cellular senescence (Lee et al., 2006). Normally, the lysosomal beta‐galactosidase displays peak activity of hydrolysing the beta‐galactosides between pH 4.0 and 4.5. In senescent cells, due to the accumulation of the enzyme, such activity is detectable at pH 6.0 (Dimri et al., 1995). This biomarker was first described in senescent human cells in vitro and in ageing skin in vivo through histochemical staining. Despite being used in many senescence studies, SA‐β‐gal activity cannot be used alone, since its activity at pH 6.0 also occurs in immortal cell lines or quiescent primary cells upon high confluency or serum starvation (Cho & Hwang, 2012). As mentioned before, multiple different measurements are needed to establish the senescent status of a cell.

Enhanced SA‐β‐gal activity has been observed in immune cells under different physiological conditions. In murine secondary lymphoid organs, memory‐phenotype (CD44^high^) T cells show increased SA‐β‐gal activity in aged mice when compared to their naïve counterparts (Shimatani et al., 2009). Injection of senescent fibroblasts into mouse peritoneal cavity leads to p16^INK4a+^ macrophages recruitment with high SA‐β‐gal expression (Liu et al., 2019). Detection of SA‐β‐gal activity by chemogenic and fluorogenic substrates is either performed by histology on fixed tissues or by flow cytometry, allowing its measurement at both population and single‐cell level. C_12_FDG (5‐dodecanoylaminofluorescein di‐β‐D‐galactopyranoside) is a galactosidase substrate covalently modified with a 12‐carbon lipophilic moiety (Debacq‐Chainiaux et al., 2009). Once inside the cell and cleaved, the fluorescent product is incorporated into the membrane structure of the cell, where it is retained. Since the SA‐β‐gal activity needs to be detected at pH 6.0, the cells are pre‐treated with lysosomal alkalisation reagents. Most importantly, cells are still alive after the detection, enabling further characterisation of SA‐β‐gal^+^ populations through in vivo transfer. Another galactosidase probe, suitable for mass spectrometry detection through adding a tellurophene reporter group, is based on a similar mechanism (Lumba et al., 2017). One caveat of both methods is the potential physiological alterations of elevated lysosomal pH or the in vitro culture when treated with alkalisation reagents. Therefore, it might be necessary to fix cells prior to staining in certain sensitive cell types.

### 2.5 Lipofuscin

Lipofuscin is a type of autofluorescent lipopigment enriched in aged neurons, muscle and skin (Moreno‐Garcia et al., 2018). While the nature and structure of lipofuscin shows tissue specificity and temporal heterogeneity, it is universally composed of lipids, metals and misfolded proteins (Hohn et al., 2010; Terman & Brunk, 2004; Zglinicki et al., 1995). The lipopigment accumulates in lysosomes and cytosol over time which, on one hand, delineates a specific cellular senescence pattern, and on the other hand, aggravates the defects of lysosomal degradation pathways during ageing (Terman et al., 1999). The levels of lipofuscins in both peritoneal macrophages, microglia and T cells from aged mice are significantly higher than those from young (Kushwaha et al., 2018; Singh Kushwaha et al., 2018; Vida et al., 2017). To customise lipofuscin detection to immune cell populations, a novel method involving an analogue of Sudan Black B (SBB), called GL13, has been developed that stains of a variety of lipids, which together make up 20%–50% of lipofuscin aggregate. With enhanced sensitivity through coupling with biotin, it enables its detection using anti‐biotin antibodies conjugated with fluorescent dyes. This allows the lipofuscins to be identified in cells using both microscopy and flow cytometry.

## 3 HALLMARKS OF CELLULAR AGEING

### 3.1 Cell stress and damage

Cells face continuous cellular damage throughout their lifetime. To guarantee homeostasis, cells evolve multiple maintenance mechanisms, which respond timely to various forms of stress and activate pathways to repair damage. However, the age‐related decline of repair mechanisms causes accumulation of damage, acting as a main driving force of cellular ageing (Harman et al., 1983; Schieber & Chandel, 2014). While it is accepted that naïve and memory T cells and tissue‐resident macrophages are generally considered to be long‐lived, and have a high chance to accumulate damage over lifetime, it is less clear whether long‐lived immune cells escape senescence or whether all senescent immune cells undergo apoptosis. However, damage can also be found in short‐lived cells, such as neutrophils and monocytes that may only survive for 1–2 days. The mechanisms that culminate in a short‐lived cell exhibiting an aged phenotype are yet to be elucidated, but they might involve inheritance of damage from their longer‐lived progenitors, which can undergo asymmetric cell division. Thus, in this section the term cellular ageing will be used to refer to the functional status of the cells that exhibit inefficient repair mechanisms, as a result of accumulated damage and stress, which is not necessarily a result of longer lifespan.

#### 3.1.1 Oxidative stress and mitochondrial dysfunction

Reactive oxygen species (ROS) refer to a class of radical and non‐radical oxygen species. The majority of cellular ROS (cROS) are generated endogenously through mitochondrial aerobic respiration (Schieber & Chandel, 2014). Meanwhile, other environmental stimuli, such as growth factors, inflammatory cytokines and ionising radiation, can also induce cROS, some of which are needed for signalling. Excessive levels of these chemically‐reactive species cause oxidative damage to various macromolecules, including DNA, proteins, and lipids. It is crucial for the cell to tightly regulate cROS levels and maintain the redox homeostasis through several antioxidant systems. However, during ageing, cellular oxidant/antioxidant ratio becomes unbalanced, usually in favour of oxidants and excessive ROS, which impairs cell homeostasis and function (Harman et al., 1983).

As mitochondrial ATP production in the electron transport chain is the main producer of cROS, mitochondrial DNA (mtDNA) and proteins are especially vulnerable to ROS (Sandalio et al., 2013). Indeed, age‐associated increase of mtDNA mutations has been found in multiple tissues (Sun et al., 2016). It impairs the normal function and integrity of mitochondria, inducing more ROS production and their leakage into the cytosol. The positive feedback loop of oxidative stress leads to cellular ageing over time. To systematically describe the mitochondrial changes occurring during ageing, mROS and mitochondrial membrane potential (MMP) are usually tested.

T‐cell activation and differentiation involves metabolic reprogramming. It is necessary to distinguish among T‐cell subsets to accurately compare oxidative stress and mitochondrial quality between old and young. Our group previously demonstrated that mROS levels significantly correlate with ageing in human naïve and central memory CD8^+^ T cells (Sanderson & Simon, 2017). In line with this, antibody‐secreting cells from old donors display an increase in mROS (Kurupati et al., 2019). Among innate immune cells, the classical monocytes isolated from human peripheral blood show no changes in mROS or MMP during ageing (Pence & Yarbro, 2018). Similarly, mROS production in aged murine neutrophils does not differ from their young counterparts (Uhl et al., 2016). However, aged bone marrow‐derived and tissue‐resident macrophages are susceptible to the accumulation of intracellular ROS (Sebastian et al., 2009). In summary, aged immune cells do not necessarily display the same mitochondrial phenotypes, requiring its detection in a cell type‐specific manner.

Oxidative stress and mitochondrial dysfunction can be assessed by fluorescent probes compatible with flow cytometry. To detect ROS, several probes are commercially available, including the H_2_‐DCFDA and CellROX reagents (Zhang et al., 2018). After passively diffusing into the cell, H_2_‐DCFDA, with its acetate groups cleaved by intracellular enzymes, undergoes oxidation by ROS and turns into a fluorescent compound (Zhu et al., 1994). One caveat of the dye is its conversion to fluorescent product accelerates in the presence of serum, heme and metalloporphyrins (Korystov et al., 2007; Ohashi et al., 2002). CellROX is available in a series of commercial fluorogenic dyes with different emission spectra. However, due to its unclear structure, reaction chemistry and kinetics, CellROX reagents may not be easily applicable to detect oxidative stress (Cheng et al., 2018).

Mito‐SOX Red has routinely been used to determine mROS levels (Zielonka et al., 2008). It can be oxidised by mitochondrial $O_{2}^{\cdot ‐}$ and yields a fluorescent oxidation product. However, high concentration of Mito‐SOX can impair mitochondrial function, also leading to the cytosolic localisation of the probe, undermining its mitochondrial specificity (Robinson et al., 2006). Thus, it is necessary to titrate its working concentration before use. Moreover, the dye is light‐sensitive and prone to auto‐oxidation, which may compromise the accuracy of superoxide detection by flow cytometry or confocal microscopy (Zielonka et al., 2008).

MMP is the driving force for mitochondrial ATP synthesis. It reflects the quality of mitochondria since depolarisation impairs mitochondrial function. The use of several common fluorescent dyes, including tetramethylrhodamine ethyl ester (TMRE), 5,5′,6,6′‐tetrachloro‐1,1′,3,3′‐tetraethylbenzimi‐dazolylcarbocyanine iodide (JC‐1), Rhodamine 123 and 3,3′‐dihexyloxacarbocyanine iodide (DioC6), facilitates the monitoring of MMP (Perry et al., 2011). These dyes are all lipophilic cationic and accumulate within mitochondria in inverse proportion to MMP. The comparison of their advantages and pitfalls has been previously summarised (Perry et al., 2011). Note that both mROS and MMP level need to be normalised with MitoTracker Green (MTG), a fluorescent probe to measure total mitochondrial mass (Kurupati et al., 2019).

#### 3.1.2 DNA damage

Cells are constantly exposed to DNA damaging agents, including chemical mutagens, irradiation and oxidative stress (Jackson & Bartek, 2009). To maintain genome stability, an evolutionary conserved pathway known as DNA damage response (DDR) can sense DNA damage and recruit the repair machineries to fix the DNA lesion. DDR mediates transient checkpoint through activating the p53‐p21^CIP^ signalling pathway before the removal of DNA damage (Ou & Schumacher, 2018). However, if DNA damage is persistent, cells may undergo apoptosis or enter cellular senescence. It can be inferred that some senescent cells may contain more DNA lesions and higher DDR level than non‐senescent ones.

8‐Oxoguanine (8‐oxoG) is one of the most common DNA lesions resulting from guanine being modified by ROS. During ageing, 8‐oxoG accumulates in various mammalian tissues (Nie et al., 2013). As mitochondrial DNA is more vulnerable to oxidative stress due to its structure and location, it displays increased 8‐oxoG both in vivo and in vitro (Barreau et al., 1996; Mecocci et al., 1993). Mice deficient in 8‐oxoG‐repair enzymes show high level of 8‐oxoG in mitochondrial DNA, which is linked with the onset of neurodegenerative diseases (Leon et al., 2016). Quantitation of cellular 8‐oxoG level can be achieved through antibody staining and followed by flow cytometry.

DNA damage also gives rise to epigenetic changes. H2AX is a minor histone H2A variant and detection of H2AX foci can serve as a readout for DNA double‐strand breaks, as well as genomic instability and telomere dysfunction (Mah et al., 2010; Nagelkerke & Span, 2016). Several kinases mediate the phosphorylation of its Ser‐139 residue and the formation of γH2AX foci at the DNA break site. γH2AX modifications can increase DNA accessibility, further enhancing the recruitment and accumulation of certain DDR proteins. Since each DNA break corresponds to one γH2AX focus (Sedelnikova et al., 2002), and, unlike other repair proteins, γH2AX is formed *de novo* upon DNA damage (Ayoub et al., 2008), it can serve as a robust biomarker reflecting the increased level of DNA damage during ageing. The number of γH2AX foci increases in human hematopoietic stem cells and progenitor cells with ageing (Quinn et al., 2018). Mouse virtual memory T cells, which are considered to be the senescent population, also show higher γH2AX level than other populations (Phadwal et al., 2012). To detect cellular γH2AX levels with high sensitivity and reliability in immune cells, we adapted the traditional immunofluorescence‐based assay to imaging flow cytometry, a technique that combines the speed of a traditional flow cytometer with the resolution and imaging of a microscope (Phadwal et al., 2012). It enables the high‐throughput analysis of γH2AX signal as well as the visualisation of its foci, which guarantees the staining specificity, for example, in human T cells (Phadwal et al., 2012).

## 4 PROTEOSTASIS

### 4.1 ER stress

The endoplasmic reticulum hosts the unfolded protein response (UPR), an important stress triggered pathway that enables the suppression of protein aggregation (Walter & Ron, 2011), and promotes cell homeostasis (Hetz et al., 2015). The orchestrated activation of three sensors is key to the UPR: the dual kinase/endoribonuclease inositol‐requiring enzyme 1α (IRE1α), the kinase PKR‐like ER‐resident kinase (PERK), and activating transcription factor 6α (ATF6α). Their activation follows the detachment of the binding immunoglobulin protein (BiP, GRP‐78), a heat shock protein 70 chaperone, and synergistically culminates in increased chaperone activity, translation inhibition and improved proteostasis (Bertolotti et al., 2000; Ye, 2000). Proteostasis relies on the activation of degradation and recycling mechanisms downstream of the UPR, such as the ubiquitin‐proteasome system and autophagy, which determines cell death or survival (Hetz et al., 2012, 2015).

Ageing is accompanied by a dysregulation of the UPR, which contributes to loss of proteostasis (Martinez et al., 2017). It has been observed in rodent tissue that the expression and activity of key chaperones, such as BiP, declines during ageing, which facilitates the accumulation of improperly folded proteins, and the formation of protein aggregates (Paz Gavilan et al., 2006). Thus, detection of low BiP levels (both mRNA or protein) may be an important tool to molecularly identify immune cell senescence. Lower levels of BiP also elicit UPR activation, which culminates in impaired protein translation. Hence, ER stress can be assessed by measuring nascent proteins with O‐propargyl‐puromycin (OPP) assays by flow cytometry (Zhang et al., 2019).

#### 4.1.1 Ubiquitin‐proteasome system

The ubiquitin‐proteasome system (UPS) acts as a major component of the intracellular protein catabolic pathway. Through the removal of misfolded or damaged proteins, it helps to maintain protein homeostasis under various forms of cellular stress (Chen et al., 2011). UPS‐mediated degradation involves two stages – the ubiquitination and the proteasome‐mediated degradation. Ubiquitin tags protein substrates to deliver them to the proteasome for degradation. Proteasomes are barrel‐shaped proteolytic machineries, containing 20S ‘core’ catalytic chambers, and 19S ‘cap’ regulatory particles, which bind the polyubiquitinated substrates and translocate them into the central chamber (Kimura et al., 2015). The most frequent form of the 20S core is the constitutive proteasome. In immune cells, a special form of proteasome, called the immunoproteasome, plays a crucial role in digesting intracellular proteins of viral origins (Nathan et al., 2013). It helps to produce small peptides for further MHC‐I‐mediated antigen presentation.

Proteasome activity declines progressively in aged mammalian cell (Carrard et al., 2003). Overexpressing proteasomal subunits has been shown to promote proteostasis and longevity in various organisms (Chondrogianni et al., 2015; Nguyen et al., 2019). Aged human lymphocytes display decreased proteasome function, mainly due to the post‐translational modification of the proteasome subunits (glycation, lipid peroxidation product conjugation and ubiquitination) (Carrard et al., 2003). Moreover, TCR‐stimulated aged CD4^+^ T cells also fail to induce transcriptional upregulation of proteasome subunit genes (Arata et al., 2019). In T cells from elderly donors, decreased proteasome activity leads to compromised IκBa degradation upon TNFα stimulation, increasing NF‐κB signalling and IL2R upregulation (Ponnappan et al., 1999).

Methods to detect the activity of different UPS components, including the proteasome, E3 ubiquitin ligases and deubiquitinating enzymes (DUBs), have been comprehensively summarised previously (Melvin et al., 2013). Here, we focus on the technique suitable for the study of primary immune cells. Fluorescently labelled activity‐based probes (ABPs) are a class of chemical compounds with three domains – a reactive group, a tri‐ or tetrapeptide recognition element and a reporter tag (Hewings et al., 2017). Once entering the proteasomal catalytic chamber, ABPs can be covalently linked to the active‐site Thr residues, detectable by flow cytometry or fluorescent microscopy. Existing subunit‐selective ABPs can distinguish all six types of constitutive and immunoproteasome subunits, therefore allowing to profile the changes in each subunit during ageing. While this is limited by the fact it inhibits the catalytic function irreversibly, ABP is still a powerful tool to measure the proteasomal activity in intact cells without the necessity of transgene or transfection (Schipper‐Krom et al., 2019).

#### 4.1.2 Autophagy

Autophagy is a conserved lysosome‐dependent degradation pathway in eukaryotic cells. There are three types of autophagy – macroautophagy, microautophagy and chaperone‐mediated autophagy (CMA). We focus on macroautophagy, the most prevalent form (hereafter referred to as autophagy) (Feng et al., 2014) and CMA, as both are closely related to ageing. Autophagy is a crucial homeostatic regulator of cell metabolism, differentiation and cell growth, balancing cell survival and death. During ageing, autophagic activity declines across multiple tissues, as is the case for macrophages, T and B lymphocytes (Bharath et al., 2020; Puleston et al., 2014; Stranks et al., 2015; ; Zhang et al., 2019). Several anti‐ageing interventions, including behavioural (calorie restriction or exercise) or pharmacological (reducing TOR or insulin/insulin‐like growth factor signalling, activating AMPK or sirtuins) converge in the induction of autophagy (Cabo et al., 2014). Possible mechanisms of how autophagy delays ageing involve the removal of protein aggregates or damaged mitochondria, prevention of stem cell attrition and oncogenic transformation, and the reduction of inflammatory responses (Rubinsztein et al., 2011). Therefore, autophagy acts both as a biomarker of ageing, and as a popular anti‐ageing target. Similarly, CMA activity declines during ageing. In mouse T cells, CMA is crucial to TCR‐mediated activation through degrading inhibitory molecules, whereas its compromised capacity during ageing negatively impact the TCR response (Valdor, 2014).

Autophagy requires core molecular machineries composed of different autophagy‐related proteins (Feng et al., 2014). It starts from a double‐membrane structure, and as this elongates, it either engulfs bulk cytosolic content or selectively sequesters a large spectrum of cargoes (Gatica et al., 2018). LC3/GABARAP (Atg8) family proteins play a crucial role in the selectivity. LC3‐II, the lipid‐modified form of LC3, is anchored into the double membranes and recruits autophagy receptors, which carries polyubiquitinated autophagic cargos (Kabeya et al., 2000). The autophagosome together with its cargo is delivered to the lysosome for degradation. The membrane‐bound LC3‐II is used as a readout for autophagy by microscopy, as it appears punctate, or by Western blot, as it runs at different molecular weight (Phadwal et al., 2012). In immune cells, flow cytometry or imaging is ideal as it can be combined with surface markers. As the fluorophore‐conjugated LC3 antibody detects both LC3‐I and II, we use saponin to selectively permeabilise plasma membrane and flush away the cytosolic non‐membrane bound LC3‐I. For LC3‐II levels to reflect autophagic flux, it is blocked with lysosomal inhibitors, such as chloroquine or bafilomycin A1, that prevent the fusion between autophagosome and lysosome and therefore accumulates LC3 levels over the time of treatment (Homewood et al., 1972; Mauvezin & Neufeld, 2015).

An alternative way to measure autophagic flux is by cationic amphiphilic tracer dyes. Cyto‐ID is an autofluorescent compound displaying a preference for autophagosomal membrane lipids (Niemann et al., 2001). These dyes have been widely applied to detect the autophagic flux in living cells with flow cytometry (Chan et al., 2012). However, some evidence suggests that during ageing autophagosomes fail to fuse with lysosomes and accumulate in the cell (Zhang et al., 2019). Measuring LC3‐II should be accompanied by detecting the extent of autophagosome‐lysosome fusion.

CMA substrates include proteins with KFERQ‐like motif, which enables their binding to heat shock cognate 71 kDa protein (HSC70, a cytosolic chaperone). The substrate‐chaperone complex is recruited to lysosomal surface by lysosome‐associated membrane protein type 2A (LAMP2A). LAMP2As are assembled into multimeric translocation complex to internalise the unfolded protein for intralysosomal degradation. To monitor CMA level in vivo, animals with transgenic reporters were developed. The fluorescent protein reporter, tagged with KFERQ sequence, is degraded specifically by CMA (Dong et al., 2020). Cells with high CMA level display lower fluorescent signal, which can be quantified by both microscopy or flow. Another method involves immunostaining of HSC70 and LAMP2A (Kaushik & Cuervo, 2009). Since lysosomes competent for CMA contains both molecules, the abundance of these lysosomes can indirectly reflect CMA level (Cuervo et al., 1997).

## 5 CONCLUSION AND PERSPECTIVES

The recent decade has witnessed a progression in the field of immune ageing. We have a comprehensive knowledge of the components of immune system, and how they respond in different physiological contexts. More importantly, immune ageing is at the centre of many age‐related phenotypes and diseases (Simon et al., 2015). Understanding its molecular and cellular mechanisms becomes more urgent with global population ageing, which also highlights the immense translational potential of strategies able to modulate and rescue immunosenescent phenotypes. The ongoing COVID‐19 pandemic is a vivid example. Old individuals are more susceptible to SARS‐CoV‐2 viral infection and complications accompanied by a severe cytokine storm (Liu, Li, et al., 2020). Thus, investigating ways to rejuvenate aged immune cells and promote robust and efficient immune responses in the elderly has become an urgent matter, and the reason why we believe that immune ageing research will be particularly incentivised after the pandemic. It will be important to first establish which immune cells increasingly undergo cellular senescence with age, before drawing any conclusions to which degree immune cell senescence contributes to immunosenescence in mammals.

Immune cells are one of the most accessible human samples, thus allowing easier ‘bench to bedside’ translation. It is especially relevant to advance in complex topics such as ageing. However, immune cells are very heterogeneous in phenotype and function, which requires systematic analysis at high resolution with single‐cell omics techniques to dissect certain phenomena (Goldman et al., 2019). Thanks to the recent advances of cloud computing, large amounts of data generated can be stored and processed with unprecedented speed. Artificial intelligence technology can further help to decipher hidden patterns among the large data volume and unravel more robust biomarkers of ageing. It is worth noting that one age‐related hallmark does not necessarily co‐exist with others. Some of them may appear earlier and drive cells into ageing, forming a hierarchical relation (Lopez‐Otin et al., 2013). Adaptation of traditional methods to fit the omics era and measure all hallmarks simultaneously is necessary, forming a ‘toolkit’ of ageing detection. This toolkit can be further applied for the exploration of other tissues, to reveal the diverse facets of ageing in the whole organism.

## CONFLICT OF INTEREST

None declared.

## AUTHOR CONTRIBUTIONS

D. Z and M. B. wrote the manuscript, and K. S. edited the manuscript.

## References

[acel13316-bib-0001] Agrawal, A. , Agrawal, S. , Cao, J.‐N. , Su, H. , Osann, K. , & Gupta, S. (2007). Altered innate immune functioning of dendritic cells in elderly humans: A role of phosphoinositide 3‐kinase‐signaling pathway. The Journal of Immunology, 178, 6912–6922. 10.4049/jimmunol.178.11.6912 17513740

[acel13316-bib-0002] Almeida‐Oliveira, A. , Smith‐Carvalho, M. , Porto, L. C. , Cardoso‐Oliveira, J. , Ribeiro, A. D. S. , Falcão, R. R. , Abdelhay, E. , Bouzas, L. F. , Thuler, L. C. S. , Ornellas, M. H. , & Diamond, H. R. (2011). Age‐related changes in natural killer cell receptors from childhood through old age. Human Immunology, 72, 319–329. 10.1016/j.humimm.2011.01.009 21262312

[acel13316-bib-0003] Alpert, A. , Pickman, Y. , Leipold, M. , Rosenberg‐Hasson, Y. , Ji, X. , Gaujoux, R. , Rabani, H. , Starosvetsky, E. , Kveler, K. , Schaffert, S. , Furman, D. , Caspi, O. , Rosenschein, U. , Khatri, P. , Dekker, C. L. , Maecker, H. T. , Davis, M. M. , & Shen‐Orr, S. S. (2019). A clinically meaningful metric of immune age derived from high‐dimensional longitudinal monitoring. Nature Medicine, 25, 487–495. 10.1038/s41591-019-0381-y PMC6686855 30842675

[acel13316-bib-0004] Arata, Y. , Watanabe, A. , Motosugi, R. , Murakami, R. , Goto, T. I. , Hori, S. , Hirayama, S. , Hamazaki, J. , & Murata, S. (2019). Defective induction of the proteasome associated with T‐cell receptor signaling underlies T‐cell senescence. Genes to Cells, 24, 801–813. 10.1111/gtc.12728 31621149

[acel13316-bib-0005] Aubert, G. , Baerlocher, G. M. , Vulto, I. , Poon, S. S. , & Lansdorp, P. M. (2012). Collapse of telomere homeostasis in hematopoietic cells caused by heterozygous mutations in telomerase genes. PLoS Genetics, 8, e1002696 10.1371/journal.pgen.1002696 22661914 PMC3355073

[acel13316-bib-0006] Aviv, A. (2020). Telomeres and COVID‐19. The FASEB Journal, 34, 7247–7252. 10.1096/fj.202001025 32427393 PMC7276714

[acel13316-bib-0007] Ayoub, N. , Jeyasekharan, A. D. , Bernal, J. A. , & Venkitaraman, A. R. (2008). HP1‐beta mobilization promotes chromatin changes that initiate the DNA damage response. Nature, 453, 682–686. 10.1038/nature06875 18438399

[acel13316-bib-0008] Baerlocher, G. M. , Vulto, I. , de Jong, G. , & Lansdorp, P. M. (2006). Flow cytometry and FISH to measure the average length of telomeres (flow FISH). Nature Protocols, 1, 2365–2376. 10.1038/nprot.2006.263 17406480

[acel13316-bib-0009] Baker, D. J. , Wijshake, T. , Tchkonia, T. , LeBrasseur, N. K. , Childs, B. G. , van de Sluis, B. , Kirkland, J. L. , & van Deursen, J. M. (2011). Clearance of p16Ink4a‐positive senescent cells delays ageing‐associated disorders. Nature, 479(7372), 232–236. 10.1038/nature10600 22048312 PMC3468323

[acel13316-bib-0010] Barreau, E. , Brossas, J. Y. , Courtois, Y. , & Treton, J. A. (1996). Accumulation of mitochondrial DNA deletions in human retina during aging. Investigative Ophthalmology & Visual Science, 37, 384–391. 8603843

[acel13316-bib-0011] Basisty, N. , Kale, A. , Jeon, O. H. , Kuehnemann, C. , Payne, T. , Rao, C. , Holtz, A. , Shah, S. , Sharma, V. , Ferrucci, L. , Campisi, J. , & Schilling, B. (2020). A proteomic atlas of senescence‐associated secretomes for aging biomarker development. PLoS Biology, 18, e3000599 10.1371/journal.pbio.3000599 31945054 PMC6964821

[acel13316-bib-0012] Bernadotte, A. , Mikhelson, V. M. , & Spivak, I. M. (2016). Markers of cellular senescence. Telomere shortening as a marker of cellular senescence. Aging (Albany NY), 8, 3–11. 10.18632/aging.100871 26805432 PMC4761709

[acel13316-bib-0013] Bertolotti, A. , Zhang, Y. , Hendershot, L. M. , Harding, H. P. , & Ron, D. (2000). Dynamic interaction of BiP and ER stress transducers in the unfolded‐protein response. Nature Cell Biology, 2, 326–332. 10.1038/35014014 10854322

[acel13316-bib-0014] Bharath, L. P. , Agrawal, M. , McCambridge, G. , Nicholas, D. A. , Hasturk, H. , Liu, J. , Jiang, K. , Liu, R. , Guo, Z. , Deeney, J. , Apovian, C. M. , Snyder‐Cappione, J. , Hawk, G. S. , Fleeman, R. M. , Pihl, R. M. F. , Thompson, K. , Belkina, A. C. , Cui, L. , … Nikolajczyk, B. S. (2020). Metformin enhances autophagy and normalizes mitochondrial function to alleviate aging‐associated inflammation. Cell Metabolism, 32(1), 44–55.e6. 10.1016/j.cmet.2020.04.015 32402267 PMC7217133

[acel13316-bib-0015] Bhushan, M. et al (2002). Tumour necrosis factor‐alpha‐induced migration of human Langerhans cells: The influence of ageing. British Journal of Dermatology, 146, 32–40. 10.1046/j.1365-2133.2002.04549.x 11841364

[acel13316-bib-0016] Brenchley, J. M. , Karandikar, N. J. , Betts, M. R. , Ambrozak, D. R. , Hill, B. J. , Crotty, L. E. , Casazza, J. P. , Kuruppu, J. , Migueles, S. A. , Connors, M. , Roederer, M. , Douek, D. C. , & Koup, R. A. (2003). Expression of CD57 defines replicative senescence and antigen‐induced apoptotic death of CD8+ T cells. Blood, 101, 2711–2720. 10.1182/blood-2002-07-2103 12433688

[acel13316-bib-0017] Bruce, J. L. , Hurford, R. K. Jr , Classon, M. , Koh, J. , & Dyson, N. (2000). Requirements for cell cycle arrest by p16INK4a. Molecular Cell, 6, 737–742. 11030353 10.1016/s1097-2765(00)00072-1

[acel13316-bib-0018] Butcher, S. K. et al (2001). Senescence in innate immune responses: Reduced neutrophil phagocytic capacity and CD16 expression in elderly humans. Journal of Leukocyte Biology, 70, 881–886. 11739550

[acel13316-bib-0019] Callender, L. A. , Carroll, E. C. , Beal, R. W. J. , Chambers, E. S. , Nourshargh, S. , Akbar, A. N. , & Henson, S. M. (2018). Human CD8(+) EMRA T cells display a senescence‐associated secretory phenotype regulated by p38 MAPK. Aging Cell, 17(1). 10.1111/acel.12675 PMC5770853 29024417

[acel13316-bib-0020] Carrard, G. , Dieu, M. , Raes, M. , Toussaint, O. , & Friguet, B. (2003). Impact of ageing on proteasome structure and function in human lymphocytes. International Journal of Biochemistry & Cell Biology, 35, 728–739. 10.1016/s1357-2725(02)00356-4 12672464

[acel13316-bib-0021] Chan, L. L. , Shen, D. , Wilkinson, A. R. , Patton, W. , Lai, N. , Chan, E. , Kuksin, D. , Lin, B. , & Qiu, J. (2012). A novel image‐based cytometry method for autophagy detection in living cells. Autophagy, 8, 1371–1382. 10.4161/auto.21028 22895056 PMC3442883

[acel13316-bib-0022] Channappanavar, R. , & Perlman, S. (2020). Age‐related susceptibility to coronavirus infections: Role of impaired and dysregulated host immunity. J Clin Invest, 130, 6204–6213. 10.1172/JCI144115 33085654 PMC7685716

[acel13316-bib-0023] Chen, B. , Retzlaff, M. , Roos, T. , & Frydman, J. (2011). Cellular strategies of protein quality control. Cold Spring Harb Perspect Biol, 3, a004374 10.1101/cshperspect.a004374 21746797 PMC3140689

[acel13316-bib-0024] Cheng, G. , Zielonka, M. , Dranka, B. , Kumar, S. N. , Myers, C. R. , Bennett, B. , Garces, A. M. , Dias Duarte Machado, L. G. , Thiebaut, D. , Ouari, O. , Hardy, M. , Zielonka, J. , & Kalyanaraman, B. (2018). Detection of mitochondria‐generated reactive oxygen species in cells using multiple probes and methods: Potentials, pitfalls, and the future. Journal of Biological Chemistry, 293, 10363–10380. 10.1074/jbc.RA118.003044 PMC6028982 29739855

[acel13316-bib-0025] Cheung, R. K. , & Utz, P. J. (2011). Screening: CyTOF‐the next generation of cell detection. Nature Reviews Rheumatology, 7, 502–503. 10.1038/nrrheum.2011.110 PMC3387986 21788983

[acel13316-bib-0026] Cho, S. , & Hwang, E. S. (2012). Status of mTOR activity may phenotypically differentiate senescence and quiescence. Molecules and Cells, 33, 597–604. 10.1007/s10059-012-0042-1 22570149 PMC3887751

[acel13316-bib-0027] Chondrogianni, N. , Georgila, K. , Kourtis, N. , Tavernarakis, N. , & Gonos, E. S. (2015). 20S proteasome activation promotes life span extension and resistance to proteotoxicity in *Caenorhabditis elegans* . The FASEB Journal, 29, 611–622. 10.1096/fj.14-252189 25395451 PMC4314225

[acel13316-bib-0028] Colonna‐Romano, G. , Bulati, M. , Aquino, A. , Pellicanò, M. , Vitello, S. , Lio, D. , Candore, G. , & Caruso, C. (2009). A double‐negative (IgD‐CD27‐) B cell population is increased in the peripheral blood of elderly people. Mechanisms of Ageing and Development, 130, 681–690. 10.1016/j.mad.2009.08.003 19698733

[acel13316-bib-0029] Cuervo, A. M. , Dice, J. F. , & Knecht, E. (1997). A population of rat liver lysosomes responsible for the selective uptake and degradation of cytosolic proteins. Journal of Biological Chemistry, 272, 5606–5615. 10.1074/jbc.272.9.5606 9038169

[acel13316-bib-0030] d'Adda di Fagagna, F. , Reaper, P. M. , Clay‐Farrace, L. , Fiegler, H. , Carr, P. , von Zglinicki, T. , Saretzki, G. , Carter, N. P. , & Jackson, S. P. (2003). A DNA damage checkpoint response in telomere‐initiated senescence. Nature, 426, 194–198. 10.1038/nature02118 14608368

[acel13316-bib-0031] de Cabo, R. , Carmona‐Gutierrez, D. , Bernier, M. , Hall, M. N. , & Madeo, F. (2014). The search for antiaging interventions: From elixirs to fasting regimens. Cell, 157, 1515–1526. 10.1016/j.cell.2014.05.031 24949965 PMC4254402

[acel13316-bib-0032] Debacq‐Chainiaux, F. , Erusalimsky, J. D. , Campisi, J. , & Toussaint, O. (2009). Protocols to detect senescence‐associated beta‐galactosidase (SA‐betagal) activity, a biomarker of senescent cells in culture and in vivo. Nature Protocols, 4, 1798–1806. 10.1038/nprot.2009.191 20010931

[acel13316-bib-0033] Demaria, M. , Ohtani, N. , Youssef, S. A. , Rodier, F. , Toussaint, W. , Mitchell, J. R. , Laberge, R.‐M. , Vijg, J. , Van Steeg, H. , Dollé, M. E. T. , Hoeijmakers, J. H. J. , de Bruin, A. , Hara, E. , & Campisi, J. (2014). An essential role for senescent cells in optimal wound healing through secretion of PDGF‐AA. Developmental Cell, 31, 722–733. 10.1016/j.devcel.2014.11.012 25499914 PMC4349629

[acel13316-bib-0034] Desdin‐Mico, G. , Soto‐Heredero, G. , Aranda, J. F. , Oller, J. , Carrasco, E. , Gabandé‐Rodríguez, E. , Blanco, E. M. , Alfranca, A. , Cussó, L. , Desco, M. , Ibañez, B. , Gortazar, A. R. , Fernández‐Marcos, P. , Navarro, M. N. , Hernaez, B. , Alcamí, A. , Baixauli, F. , & Mittelbrunn, M. (2020). T cells with dysfunctional mitochondria induce multimorbidity and premature senescence. Science, 368, 1371–1376. 10.1126/science.aax0860 32439659 PMC7616968

[acel13316-bib-0035] Di Mitri, D. et al (2011). Reversible senescence in human CD4+CD45RA+CD27‐ memory T cells. The Journal of Immunology, 187, 2093–2100. 10.4049/jimmunol.1100978 21788446

[acel13316-bib-0036] Dimri, G. P. , Lee, X. , Basile, G. , Acosta, M. , Scott, G. , Roskelley, C. , Medrano, E. E. , Linskens, M. , Rubelj, I. , & Pereira‐Smith, O. (1995). A biomarker that identifies senescent human cells in culture and in aging skin in vivo. Proceedings of the National Academy of Sciences of the United States of America, 92, 9363–9367. 10.1073/pnas.92.20.9363 7568133 PMC40985

[acel13316-bib-0037] Dong, S. , Aguirre‐Hernandez, C. , Scrivo, A. , Eliscovich, C. , Arias, E. , Bravo‐Cordero, J. J. , & Cuervo, A. M. (2020). Monitoring spatiotemporal changes in chaperone‐mediated autophagy in vivo. Nature Communications, 11, 645 10.1038/s41467-019-14164-4 PMC6994528 32005807

[acel13316-bib-0038] Dou, M. , Clair, G. , Tsai, C.‐F. , Xu, K. , Chrisler, W. B. , Sontag, R. L. , Zhao, R. , Moore, R. J. , Liu, T. , Pasa‐Tolic, L. , Smith, R. D. , Shi, T. , Adkins, J. N. , Qian, W.‐J. , Kelly, R. T. , Ansong, C. , & Zhu, Y. (2019). High‐throughput single cell proteomics enabled by multiplex isobaric labeling in a nanodroplet sample preparation platform. Analytical Chemistry, 91, 13119–13127. 10.1021/acs.analchem.9b03349 31509397 PMC7192326

[acel13316-bib-0039] Drew, W. , Wilson, D. V. , & Sapey, E. (2018). Inflammation and neutrophil immunosenescence in health and disease: Targeted treatments to improve clinical outcomes in the elderly. Experimental Gerontology, 105, 70–77. 10.1016/j.exger.2017.12.020 29288715

[acel13316-bib-1004] Durdik, M. , Kosik, P. , Gursky, J. , Vokalova, L. , Markova, E. , & Belyaev, I. (2015). Imaging flow cytometry as a sensitive tool to detect low‐dose‐induced DNA damage by analyzing 53BP1 and gammaH2AX foci in human lymphocytes. Cytometry A, 87(12), 1070–1078. 10.1002/cyto.a.22731 26243567

[acel13316-bib-0040] Enge, M. , Arda, H. E. , Mignardi, M. , Beausang, J. , Bottino, R. , Kim, S. K. , & Quake, S. R. (2017). Single‐cell analysis of human pancreas reveals transcriptional signatures of aging and somatic mutation patterns. Cell, 171(2), 321–330.e14. 10.1016/j.cell.2017.09.004 28965763 PMC6047899

[acel13316-bib-0041] Ergen, A. V. , Boles, N. C. , & Goodell, M. A. (2012). Rantes/Ccl5 influences hematopoietic stem cell subtypes and causes myeloid skewing. Blood, 119, 2500–2509. 10.1182/blood-2011-11-391730 22289892 PMC3311273

[acel13316-bib-0042] Fali, T. , Papagno, L. , Bayard, C. , Mouloud, Y. , Boddaert, J. , Sauce, D. , & Appay, V. (2019). New Insights into lymphocyte differentiation and aging from telomere length and telomerase activity measurements. The Journal of Immunology, 202, 1962–1969. 10.4049/jimmunol.1801475 30737273

[acel13316-bib-0043] Farr, J. N. , Fraser, D. G. , Wang, H. , Jaehn, K. , Ogrodnik, M. B. , Weivoda, M. M. , Drake, M. T. , Tchkonia, T. , LeBrasseur, N. K. , Kirkland, J. L. , Bonewald, L. F. , Pignolo, R. J. , Monroe, D. G. , & Khosla, S. (2016). Identification of senescent cells in the bone microenvironment. Journal of Bone and Mineral Research, 31, 1920–1929. 10.1002/jbmr.2892 27341653 PMC5289710

[acel13316-bib-0044] Feng, Y. , He, D. , Yao, Z. , & Klionsky, D. J. (2014). The machinery of macroautophagy. Cell Research, 24, 24–41. 10.1038/cr.2013.168 24366339 PMC3879710

[acel13316-bib-0045] Franceschi, C. , Bonafè, M. , Valensin, S. , Olivieri, F. , De luca, M. , Ottaviani, E. , & De benedictis, G. (2000). Inflamm‐aging. An evolutionary perspective on immunosenescence. Annals of the New York Academy of Sciences, 908, 244–254. 10.1111/j.1749-6632.2000.tb06651.x 10911963

[acel13316-bib-0046] Franceschi, C. , Garagnani, P. , Parini, P. , Giuliani, C. , & Santoro, A. (2018). Inflammaging: A new immune‐metabolic viewpoint for age‐related diseases. Nature Reviews Endocrinology, 14, 576–590. 10.1038/s41574-018-0059-4 30046148

[acel13316-bib-0047] Franceschi, C. , Garagnani, P. , Vitale, G. , Capri, M. , & Salvioli, S. (2017). Inflammaging and ‘Garb‐aging'. Trends in Endocrinology and Metabolism, 28, 199–212. 10.1016/j.tem.2016.09.005 27789101

[acel13316-bib-0048] Frasca, D. (2018). Senescent B cells in aging and age‐related diseases: Their role in the regulation of antibody responses. Experimental Gerontology, 107, 55–58. 10.1016/j.exger.2017.07.002 28687479 PMC5754260

[acel13316-bib-0049] Frasca, D. , Diaz, A. , Romero, M. , & Blomberg, B. B. (2017). Human peripheral late/exhausted memory B cells express a senescent‐associated secretory phenotype and preferentially utilize metabolic signaling pathways. Experimental Gerontology, 87, 113–120. 10.1016/j.exger.2016.12.001 27931848

[acel13316-bib-0050] Fulop, T. , Dupuis, G. , Witkowski, J. M. , & Larbi, A. (2016). The role of immunosenescence in the development of age‐related diseases. Revista De Investigacion Clinica, 68, 84–91. 27103044

[acel13316-bib-0051] Gatica, D. , Lahiri, V. , & Klionsky, D. J. (2018). Cargo recognition and degradation by selective autophagy. Nature Cell Biology, 20, 233–242. 10.1038/s41556-018-0037-z 29476151 PMC6028034

[acel13316-bib-0052] Goldman, S. L. , MacKay, M. , Afshinnekoo, E. , Melnick, A. M. , Wu, S. , & Mason, C. E. (2019). The impact of heterogeneity on single‐cell sequencing. Front Genet, 10, 8 10.3389/fgene.2019.00008 30881372 PMC6405636

[acel13316-bib-0053] Gorgoulis, V. , Adams, P. D. , Alimonti, A. , Bennett, D. C. , Bischof, O. , Bishop, C. , Campisi, J. , Collado, M. , Evangelou, K. , Ferbeyre, G. , Gil, J. , Hara, E. , Krizhanovsky, V. , Jurk, D. , Maier, A. B. , Narita, M. , Niedernhofer, L. , Passos, J. F. , … Demaria, M. (2019). Cellular senescence: Defining a path forward. Cell, 179, 813–827. 10.1016/j.cell.2019.10.005 31675495

[acel13316-bib-0054] Group R. C. et al (2020). Dexamethasone in hospitalized patients with Covid‐19 ‐ preliminary report. New England Journal of Medicine, 2020, 10.1056/NEJMoa2021436

[acel13316-bib-0055] Hall, B. M. et al (2016). Aging of mice is associated with p16(Ink4a)‐ and beta‐galactosidase‐positive macrophage accumulation that can be induced in young mice by senescent cells. Aging (Albany NY), 8, 1294–1315. 10.18632/aging.100991 27391570 PMC4993332

[acel13316-bib-0056] Hao, Y. , O'Neill, P. , Naradikian, M. S. , Scholz, J. L. , & Cancro, M. P. (2011). A B‐cell subset uniquely responsive to innate stimuli accumulates in aged mice. Blood, 118, 1294–1304. 10.1182/blood-2011-01-330530 21562046 PMC3152496

[acel13316-bib-0057] Harman, D. (1983). Free radical theory of aging: Consequences of mitochondrial aging. Ageing Research Reviews, 6, 1983.

[acel13316-bib-0058] He, G. , Siddik, Z. H. , Huang, Z. , Wang, R. , Koomen, J. , Kobayashi, R. , Khokhar, A. R. , & Kuang, J. (2005). Induction of p21 by p53 following DNA damage inhibits both Cdk4 and Cdk2 activities. Oncogene, 24, 2929–2943. 10.1038/sj.onc.1208474 15735718

[acel13316-bib-0059] Hearps, A. C. , Martin, G. E. , Angelovich, T. A. , Cheng, W.‐J. , Maisa, A. , Landay, A. L. , Jaworowski, A. , & Crowe, S. M. (2012). Aging is associated with chronic innate immune activation and dysregulation of monocyte phenotype and function. Aging Cell, 11, 867–875. 10.1111/j.1474-9726.2012.00851.x 22708967

[acel13316-bib-0060] Henson, S. M. , & Akbar, A. N. (2009). KLRG1–more than a marker for T cell senescence. Age (Dordr), 31, 285–291. 10.1007/s11357-009-9100-9 19479342 PMC2813054

[acel13316-bib-0061] Henson, S. M. , Franzese, O. , Macaulay, R. , Libri, V. , Azevedo, R. I. , Kiani‐Alikhan, S. , Plunkett, F. J. , Masters, J. E. , Jackson, S. , Griffiths, S. J. , Pircher, H.‐P. , Soares, M. V. D. , & Akbar, A. N. (2009). KLRG1 signaling induces defective Akt (ser473) phosphorylation and proliferative dysfunction of highly differentiated CD8+ T cells. Blood, 113, 6619–6628. 10.1182/blood-2009-01-199588 19406987

[acel13316-bib-0062] Hetz, C. (2012). The unfolded protein response: Controlling cell fate decisions under ER stress and beyond. Nature Reviews Molecular Cell Biology, 13, 89–102. 10.1038/nrm3270 22251901

[acel13316-bib-0063] Hetz, C. , Chevet, E. , & Oakes, S. A. (2015). Proteostasis control by the unfolded protein response. Nature Cell Biology, 17, 829–838. 10.1038/ncb3184 26123108 PMC5546321

[acel13316-bib-0064] Hewings, D. S. , Flygare, J. A. , Wertz, I. E. , & Bogyo, M. (2017). Activity‐based probes for the multicatalytic proteasome. FEBS Journal, 284, 1540–1554. 10.1111/febs.14016 28107776

[acel13316-bib-0065] Hiyama, K. et al (1995). Activation of telomerase in human lymphocytes and hematopoietic progenitor cells. The Journal of Immunology, 155, 3711–3715. 7561072

[acel13316-bib-0066] Hohn, A. , Jung, T. , Grimm, S. , & Grune, T. (2010). Lipofuscin‐bound iron is a major intracellular source of oxidants: Role in senescent cells. Free Radical Biology and Medicine, 48, 1100–1108. 10.1016/j.freeradbiomed.2010.01.030 20116426

[acel13316-bib-0067] Homewood, C. A. , Warhurst, D. C. , Peters, W. , & Baggaley, V. C. (1972). Lysosomes, pH and the anti‐malarial action of chloroquine. Nature, 235, 50–52. 10.1038/235050a0 4550396

[acel13316-bib-0068] Huang, E. E. , Tedone, E. , O’Hara, R. , Cornelius, C. , Lai, T.‐P. , Ludlow, A. , Wright, W. E. , & Shay, J. W. (2017). The maintenance of telomere length in CD28+ T cells during T lymphocyte stimulation. Scientific Reports, 7, 6785 10.1038/s41598-017-05174-7 28754961 PMC5533788

[acel13316-bib-0069] Jackson, S. P. , & Bartek, J. (2009). The DNA‐damage response in human biology and disease. Nature, 461, 1071–1078. 10.1038/nature08467 19847258 PMC2906700

[acel13316-bib-0070] Judge, S. J. , Murphy, W. J. , & Canter, R. J. (2020). Characterizing the dysfunctional NK cell: Assessing the clinical relevance of exhaustion, anergy, and senescence. Frontiers in Cellular and Infection Microbiology, 10, 49 10.3389/fcimb.2020.00049 32117816 PMC7031155

[acel13316-bib-0071] Kabeya, Y. (2000). LC3, a mammalian homologue of yeast Apg8p, is localized in autophagosome membranes after processing. EMBO Journal, 19, 5720–5728. 10.1093/emboj/19.21.5720 PMC305793 11060023

[acel13316-bib-0072] Kapasi, A. A. , & Singhal, P. C. (1999). Aging splenocyte and thymocyte apoptosis is associated with enhanced expression of p53, bax, and caspase‐3. Molecular Cell Biology Research Communications, 1, 78–81. 10.1006/mcbr.1999.0106 10329482

[acel13316-bib-0073] Kaushik, S. , & Cuervo, A. M. (2009). Methods to monitor chaperone‐mediated autophagy. Methods in Enzymology, 452, 297–324. 10.1016/S0076-6879(08)03619-7 19200890 PMC4300957

[acel13316-bib-0074] Kennedy, B. K. , Berger, S. L. , Brunet, A. , Campisi, J. , Cuervo, A. M. , Epel, E. S. , Franceschi, C. , Lithgow, G. J. , Morimoto, R. I. , Pessin, J. E. , Rando, T. A. , Richardson, A. , Schadt, E. E. , Wyss‐Coray, T. , & Sierra, F. (2014). Geroscience: Linking aging to chronic disease. Cell, 159, 709–713. 10.1016/j.cell.2014.10.039 25417146 PMC4852871

[acel13316-bib-0075] Kimura, H. , Caturegli, P. , Takahashi, M. , & Suzuki, K. (2015). New insights into the function of the immunoproteasome in immune and nonimmune cells. Journal of Immunology Research, 2015, 541984 10.1155/2015/541984 26636107 PMC4617869

[acel13316-bib-0076] Kloc, M. , Ghobrial, R. M. , & Kubiak, J. Z. (2020). The role of genetic sex and mitochondria in response to COVID‐19 infection. International Archives of Allergy and Immunology, 181, 629–634. 10.1159/000508560 32564017 PMC7360490

[acel13316-bib-0077] Korystov, Y. N. , Shaposhnikova, V. V. , Korystova, A. F. , & Emel'yanov, M. O. (2007). Detection of reactive oxygen species induced by radiation in cells using the dichlorofluorescein assay. Radiation Research, 168, 226–232. 10.1667/RR0925.1 17638409

[acel13316-bib-0078] Kurupati, R. K. , Haut, L. H. , Schmader, K. E. , & Ertl, H. C. (2019). Age‐related changes in B cell metabolism. Aging (Albany NY), 11, 4367–4381. 10.18632/aging.102058 31283526 PMC6660053

[acel13316-bib-0079] Kushwaha, S. S. , Patro, N. , & Patro, I. K. (2018). A sequential study of age‐related lipofuscin accumulation in hippocampus and striate cortex of rats. Annals of Neurosciences, 25(7372), 223–233. 31000961 10.1159/000490908 PMC6470335

[acel13316-bib-0080] Laberge, R. M. , Sun, Y. , Orjalo, A. V. , Patil, C. K. , Freund, A. , Zhou, L. , Curran, S. C. , Davalos, A. R. , Wilson‐Edell, K. A. , Liu, S. , Limbad, C. , Demaria, M. , Li, P. , Hubbard, G. B. , Ikeno, Y. , Javors, M. , Desprez, P.‐Y. , Benz, C. C. , Kapahi, P. , … Campisi, J. (2015). MTOR regulates the pro‐tumorigenic senescence‐associated secretory phenotype by promoting IL1A translation. Nature Cell Biology, 17, 1049–1061. 10.1038/ncb3195 26147250 PMC4691706

[acel13316-bib-0081] Lanier, L. L. (2013). Shades of grey–the blurring view of innate and adaptive immunity. Nature Reviews Immunology, 13, 73–74. 10.1038/nri3389 23469373

[acel13316-bib-0082] Lanna, A. , Henson, S. M. , Escors, D. , & Akbar, A. N. (2014). The kinase p38 activated by the metabolic regulator AMPK and scaffold TAB1 drives the senescence of human T cells. Nature Immunology, 15, 965–972. 10.1038/ni.2981 25151490 PMC4190666

[acel13316-bib-0083] Le Page, A. , Dupuis, G. , Larbi, A. , Witkowski, J. M. , & Fulop, T. (2018). Signal transduction changes in CD4(+) and CD8(+) T cell subpopulations with aging. Experimental Gerontology, 105, 128–139. 10.1016/j.exger.2018.01.005 29307735

[acel13316-bib-0084] Lee, B. Y. et al (2006). Senescence‐associated beta‐galactosidase is lysosomal beta‐galactosidase. Aging Cell, 5, 187–195. 10.1111/j.1474-9726.2006.00199.x 16626397

[acel13316-bib-0085] Leon, J. , Sakumi, K. , Castillo, E. , Sheng, Z. , Oka, S. , & Nakabeppu, Y. (2016). 8‐Oxoguanine accumulation in mitochondrial DNA causes mitochondrial dysfunction and impairs neuritogenesis in cultured adult mouse cortical neurons under oxidative conditions. Scientific Reports, 6, 22086 10.1038/srep22086 26912170 PMC4766534

[acel13316-bib-0086] Lin, Y. et al (2015). Age‐associated telomere attrition of lymphocytes in vivo is co‐ordinated with changes in telomerase activity, composition of lymphocyte subsets and health conditions. Clinical Science (Lond), 128, 367–377. 10.1042/CS20140481 PMC5421624 25317735

[acel13316-bib-0087] Liu, D. , Paczkowski, P. , Mackay, S. , Ng, C. , & Zhou, J. (2020). Single‐cell multiplexed proteomics on the isolight resolves cellular functional heterogeneity to reveal clinical responses of cancer patients to immunotherapies. Methods in Molecular Biology, 2055, 413–431. 10.1007/978-1-4939-9773-2_19 31502163

[acel13316-bib-0088] Liu, J. Y. et al (2019). Cells exhibiting strong p16 (INK4a) promoter activation in vivo display features of senescence. Proceedings of the National Academy of Sciences of the United States of America, 116, 2603–2611. 10.1073/pnas.1818313116 30683717 PMC6377452

[acel13316-bib-0089] Liu, J. , Li, S. , Liu, J. , Liang, B. , Wang, X. , Wang, H. , Li, W. , Tong, Q. , Yi, J. , Zhao, L. , Xiong, L. , Guo, C. , Tian, J. , Luo, J. , Yao, J. , Pang, R. , Shen, H. , Peng, C. , Liu, T. , … Zheng, X. (2020). Longitudinal characteristics of lymphocyte responses and cytokine profiles in the peripheral blood of SARS‐CoV‐2 infected patients. EBioMedicine, 55, 102763 10.1016/j.ebiom.2020.102763 32361250 PMC7165294

[acel13316-bib-0090] Liu, L. , Bailey, S. M. , Okuka, M. , Muñoz, P. , Li, C. , Zhou, L. , Wu, C. , Czerwiec, E. , Sandler, L. , Seyfang, A. , Blasco, M. A. , & Keefe, D. L. (2007). Telomere lengthening early in development. Nature Cell Biology, 9, 1436–1441. 10.1038/ncb1664 17982445

[acel13316-bib-0091] Liu, Y. , Johnson, S. M. , Fedoriw, Y. , Rogers, A. B. , Yuan, H. , Krishnamurthy, J. , & Sharpless, N. E. (2011). Expression of p16INK4a prevents cancer and promotes aging in lymphocytes. Blood, 117, 3257–3267. 21245485 10.1182/blood-2010-09-304402 PMC3069667

[acel13316-bib-0092] Liu, Y. , Sanoff, H. K. , Cho, H. , Burd, C. E. , Torrice, C. , Ibrahim, J. G. , & Sharpless, N. E. (2009). Expression of p16INK4a in peripheral blood T‐cells is a biomarker of human aging. Aging Cell, 8, 439–448. 19485966 10.1111/j.1474-9726.2009.00489.x PMC2752333

[acel13316-bib-0093] Liu, Y. , & Sharpless, N. E. (2009). Tumor suppressor mechanisms in immune aging. Current Opinion in Immunology, 21, 431–439. 10.1016/j.coi.2009.05.011 19535234 PMC2725203

[acel13316-bib-0094] Liu, Y. , Tavana, O. , & Gu, W. (2019). p53 modifications: Exquisite decorations of the powerful guardian. J Mol Cell Biol, 11, 564–577. 10.1093/jmcb/mjz060 31282934 PMC6736412

[acel13316-bib-1007] Liu, J. , Xu, Y. , Stoleru, D. , & Salic, A. (2012). Imaging protein synthesis in cells and tissues with an alkyne analog of puromycin. Proceedings of the National Academy of Sciences, 109(2), 413–418. 10.1073/pnas.1111561108 PMC3258597 22160674

[acel13316-bib-0095] Lopez‐Otin, C. , Blasco, M. A. , Partridge, L. , Serrano, M. , & Kroemer, G. (2013). The hallmarks of aging. Cell, 153, 1194–1217. 10.1016/j.cell.2013.05.039 23746838 PMC3836174

[acel13316-bib-0096] Lopez‐Verges, S. , Milush, J. M. , Pandey, S. , York, V. A. , Arakawa‐Hoyt, J. , Pircher, H. , Norris, P. J. , Nixon, D. F. , & Lanier, L. L. (2010). CD57 defines a functionally distinct population of mature NK cells in the human CD56dimCD16+ NK‐cell subset. Blood, 116, 3865–3874. 10.1182/blood-2010-04-282301 20733159 PMC2981540

[acel13316-bib-0097] Ludlow, A. T. , Robin, J. D. , Sayed, M. , Litterst, C. M. , Shelton, D. N. , Shay, J. W. , & Wright, W. E. (2014). Quantitative telomerase enzyme activity determination using droplet digital PCR with single cell resolution. Nucleic Acids Research, 42, e104 10.1093/nar/gku439 24861623 PMC4117742

[acel13316-bib-0098] Lumba, M. A. et al (2017). A beta‐galactosidase probe for the detection of cellular senescence by mass cytometry. Organic & Biomolecular Chemistry, 15, 6388–6392. 10.1039/c7ob01227f 28726964

[acel13316-bib-0099] Luo, Y. , Zou, P. , Zou, J. , Wang, J. , Zhou, D. , & Liu, L. (2011). Autophagy regulates ROS‐induced cellular senescence via p21 in a p38 MAPKα dependent manner. Experimental Gerontology, 46, 860–867. 21816217 10.1016/j.exger.2011.07.005 PMC3390188

[acel13316-bib-0100] Lurie, N. , Saville, M. , Hatchett, R. , & Halton, J. (2020). Developing Covid‐19 vaccines at pandemic speed. New England Journal of Medicine, 382, 1969–1973. 10.1056/NEJMp2005630 32227757

[acel13316-bib-0101] Mah, L. J. , El‐Osta, A. , & Karagiannis, T. C. (2010). gammaH2AX: A sensitive molecular marker of DNA damage and repair. Leukemia, 24, 679–686. 10.1038/leu.2010.6 20130602

[acel13316-bib-0102] Martinez, G. , Duran‐Aniotz, C. , Cabral‐Miranda, F. , Vivar, J. P. , & Hetz, C. (2017). Endoplasmic reticulum proteostasis impairment in aging. Aging Cell, 16, 615–623. 10.1111/acel.12599 28436203 PMC5506418

[acel13316-bib-0103] Mauvezin, C. , & Neufeld, T. P. (2015). Bafilomycin A1 disrupts autophagic flux by inhibiting both V‐ATPase‐dependent acidification and Ca‐P60A/SERCA‐dependent autophagosome‐lysosome fusion. Autophagy, 11, 1437–1438. 10.1080/15548627.2015.1066957 26156798 PMC4590655

[acel13316-bib-0104] McLachlan, J. A. , Serkin, C. D. , Morrey, K. M. , & Bakouche, O. (1995). Antitumoral properties of aged human monocytes. The Journal of Immunology, 154, 832–843. 7814887

[acel13316-bib-0105] Mecocci, P. , MacGarvey, U. , Kaufman, A. E. , Koontz, D. , Shoffner, J. M. , Wallace, D. C. , & Beal, M. F. (1993). Oxidative damage to mitochondrial DNA shows marked age‐dependent increases in human brain. Annals of Neurology, 34, 609–616. 10.1002/ana.410340416 8215249

[acel13316-bib-0106] Meftahi, G. H. , Jangravi, Z. , Sahraei, H. , & Bahari, Z. (2020). The possible pathophysiology mechanism of cytokine storm in elderly adults with COVID‐19 infection: The contribution of "inflame‐aging". Inflammation Research, 69, 825–839. 10.1007/s00011-020-01372-8 32529477 PMC7289226

[acel13316-bib-0107] Melvin, A. T. , Woss, G. S. , Park, J. H. , Waters, M. L. , & Allbritton, N. L. (2013). Measuring activity in the ubiquitin‐proteasome system: From large scale discoveries to single cells analysis. Cell Biochemistry and Biophysics, 67, 75–89. 10.1007/s12013-013-9621-9 23686610 PMC3758771

[acel13316-bib-0108] Mlynarczyk, C. , & Fåhraeus, R. (2014). Endoplasmic reticulum stress sensitizes cells to DNA damage‐induced apoptosis through p53‐dependent suppression of p21 CDKN1A. Nature Communications, 5, 1–16. 10.1038/ncomms6067 25295585

[acel13316-bib-0109] Mondal, A. M. , Horikawa, I. , Pine, S. R. , Fujita, K. , Morgan, K. M. , Vera, E. , Mazur, S. J. , Appella, E. , Vojtesek, B. , Blasco, M. A. , Lane, D. P. , & Harris, C. C. (2013). p53 isoforms regulate aging‐ and tumor‐associated replicative senescence in T lymphocytes. J Clin Invest, 123, 5247–5257. 10.1172/JCI70355 24231352 PMC3859419

[acel13316-bib-0110] Montalto, M. C. , Phillips, J. S. , & Ray, F. A. (1999). Telomerase activation in human fibroblasts during escape from crisis. Journal of Cellular Physiology, 180, 46–52.10.1002/(SICI)1097‐4652(199907)180:1<46:AID‐JCP5>3.0.CO;2‐K 10362016 10.1002/(SICI)1097-4652(199907)180:1<46::AID-JCP5>3.0.CO;2-K

[acel13316-bib-0111] Moreno‐Garcia, A. , Kun, A. , Calero, O. , Medina, M. , & Calero, M. (2018). An overview of the role of lipofuscin in age‐related neurodegeneration. Frontiers in Neuroscience, 12, 464 10.3389/fnins.2018.00464 30026686 PMC6041410

[acel13316-bib-0112] Nagelkerke, A. , & Span, P. N. (2016). Staining against phospho‐H2AX (gamma‐H2AX) as a marker for DNA damage and genomic instability in cancer tissues and cells. Advances in Experimental Medicine and Biology, 899, 1–10. 10.1007/978-3-319-26666-4_1 27325258

[acel13316-bib-1005] Naidoo, N. , Ferber, M. , Master, M. , Zhu, Y. , & Pack, A. I. (2008). Aging impairs the unfolded protein response to sleep deprivation and leads to Proapoptotic signaling. Journal of Neuroscience, 28(26), 6539–6548. 10.1523/JNEUROSCI.5685-07.2008 18579727 PMC2925257

[acel13316-bib-0113] Najarro, K. , Nguyen, H. , Chen, G. , Xu, M. , Alcorta, S. , Yao, X. , Zukley, L. , Metter, E. J. , Truong, T. , Lin, Y. , Li, H. , Oelke, M. , Xu, X. , Ling, S. M. , Longo, D. L. , Schneck, J. , Leng, S. , Ferrucci, L. , & Weng, N.‐P. (2015). Telomere length as an indicator of the robustness of B‐ and T‐cell response to influenza in older adults. Journal of Infectious Diseases, 212, 1261–1269. 10.1093/infdis/jiv202 PMC4577042 25828247

[acel13316-bib-0114] Nathan, J. A. , Spinnenhirn, V. , Schmidtke, G. , Basler, M. , Groettrup, M. , & Goldberg, A. L. (2013). Immuno‐ and constitutive proteasomes do not differ in their abilities to degrade ubiquitinated proteins. Cell, 152, 1184–1194. 10.1016/j.cell.2013.01.037 23452861 PMC3791394

[acel13316-bib-0115] Nguyen, N. N. et al .(2019). Proteasome beta5 subunit overexpression improves proteostasis during aging and extends lifespan in *Drosophila melanogaster* . Scientific Reports, 9, 3170 10.1038/s41598-019-39508-4 30816680 PMC6395709

[acel13316-bib-0116] Nie, B. , Gan, W. , Shi, F. , Hu, G.‐X. , Chen, L.‐G. , Hayakawa, H. , Sekiguchi, M. , & Cai, J.‐P. (2013). Age‐dependent accumulation of 8‐oxoguanine in the DNA and RNA in various rat tissues. Oxidative Medicine and Cellular Longevity, 2013, 1–9. 10.1155/2013/303181 PMC3657452 23738036

[acel13316-bib-0117] Niemann, A. , Baltes, J. , & Elsasser, H. P. (2001). Fluorescence properties and staining behavior of monodansylpentane, a structural homologue of the lysosomotropic agent monodansylcadaverine. Journal of Histochemistry and Cytochemistry, 49, 177–185. 10.1177/002215540104900205 11156686

[acel13316-bib-0118] Nyugen, J. , Agrawal, S. , Gollapudi, S. , & Gupta, S. (2010). Impaired functions of peripheral blood monocyte subpopulations in aged humans. Journal of Clinical Immunology, 30, 806–813. 10.1007/s10875-010-9448-8 20703784 PMC2970801

[acel13316-bib-0119] Ohashi, T. , Mizutani, A. , Murakami, A. , Kojo, S. , Ishii, T. , & Taketani, S. (2002). Rapid oxidation of dichlorodihydrofluorescin with heme and hemoproteins: Formation of the fluorescein is independent of the generation of reactive oxygen species. FEBS Letters, 511, 21–27. 10.1016/s0014-5793(01)03262-8 11821042

[acel13316-bib-0120] Olsen, L. R. , Leipold, M. D. , Pedersen, C. B. , & Maecker, H. T. (2019). The anatomy of single cell mass cytometry data. Cytometry A, 95, 156–172. 10.1002/cyto.a.23621 30277658

[acel13316-bib-0121] Onyema, O. O. et al (2012). Cellular aging and senescence characteristics of human T‐lymphocytes. Biogerontology, 13, 169–181. 10.1007/s10522-011-9366-z 22102004

[acel13316-bib-0122] Ou, H. L. , & Schumacher, B. (2018). DNA damage responses and p53 in the aging process. Blood, 131, 488–495. 10.1182/blood-2017-07-746396 29141944 PMC6839964

[acel13316-bib-0123] Pang, W. W. , Price, E. A. , Sahoo, D. , Beerman, I. , Maloney, W. J. , Rossi, D. J. , Schrier, S. L. , & Weissman, I. L. (2011). Human bone marrow hematopoietic stem cells are increased in frequency and myeloid‐biased with age. Proceedings of the National Academy of Sciences of the United States of America, 108, 20012–20017. 10.1073/pnas.1116110108 22123971 PMC3250139

[acel13316-bib-0124] Pawelec, G. , Wagner, W. , Adibzadeh, M. , & Engel, A. (1999). T cell immunosenescence in vitro and in vivo. Experimental Gerontology, 34, 419–429. 10.1016/s0531-5565(99)00002-9 10433396

[acel13316-bib-0125] Paz Gavilan, M. , Vela, J. , Castaño, A. , Ramos, B. , del Río, J. C. , Vitorica, J. , & Ruano, D. (2006). Cellular environmentfacilitates protein accumulation in aged rat hippocampus. Neurobiology of Aging, 27, 973–982. 10.1016/j.neurobiolaging.2005.05.010. 15964666

[acel13316-bib-0126] Pence, B. D. , & Yarbro, J. R. (2018). Aging impairs mitochondrial respiratory capacity in classical monocytes. Experimental Gerontology, 108, 112–117. 10.1016/j.exger.2018.04.008 29655929

[acel13316-bib-0127] Perry, S. W. , Norman, J. P. , Barbieri, J. , Brown, E. B. , & Gelbard, H. A. (2011). Mitochondrial membrane potential probes and the proton gradient: A practical usage guide. BioTechniques, 50, 98–115. 10.2144/000113610 21486251 PMC3115691

[acel13316-bib-0128] Pfeiffer, V. , & Lingner, J. (2013). Replication of telomeres and the regulation of telomerase. Cold Spring Harbor Perspectives in Biology, 5, a010405 10.1101/cshperspect.a010405 23543032 PMC3632062

[acel13316-bib-0129] Phadwal, K. , Alegre‐Abarrategui, J. , Watson, A. S. , Pike, L. , Anbalagan, S. , Hammond, E. M. , Wade‐Martins, R. , McMichael, A. , Klenerman, P. , & Simon, A. K. (2012). A novel method for autophagy detection in primary cells: Impaired levels of macroautophagy in immunosenescent T cells. Autophagy, 8, 677–689. 10.4161/auto.18935 22302009 PMC3405842

[acel13316-bib-0130] Plunkett, F. J. et al (2007). The loss of telomerase activity in highly differentiated CD8+CD28‐CD27‐ T cells is associated with decreased Akt (Ser473) phosphorylation. The Journal of Immunology, 178, 7710–7719. 10.4049/jimmunol.178.12.7710 17548608

[acel13316-bib-0131] Ponnappan, U. , Zhong, M. , & Trebilcock, G. U. (1999). Decreased proteasome‐mediated degradation in T cells from the elderly: A role in immune senescence. Cellular Immunology, 192, 167–174. 10.1006/cimm.1998.1418 10087185

[acel13316-bib-0132] Prata, L. , Ovsyannikova, I. G. , Tchkonia, T. , & Kirkland, J. L. (2018). Senescent cell clearance by the immune system: Emerging therapeutic opportunities. Seminars in Immunology, 40, 101275 10.1016/j.smim.2019.04.003 31088710 PMC7061456

[acel13316-bib-0133] Prieur, A. , Besnard, E. , Babled, A. , & Lemaitre, J. M. (2011). p53 and p16 INK4a independent induction of senescence by chromatin‐dependent alteration of S‐phase progression. Nature Communications, 2, 1–10. 10.1038/ncomms1473 21915115

[acel13316-bib-0134] Puleston, D. J. , Zhang, H. , Powell, T. J. , Lipina, E. , Sims, S. , Panse, I. , Watson, A. S. , Cerundolo, V. , Townsend, A. R. M. , Klenerman, P. , & Simon, A. K. (2014). Autophagy is a critical regulator of memory CD8(+) T cell formation. Elife, 3, 10.7554/eLife.03706 PMC4225493 25385531

[acel13316-bib-0135] Qian, F. , Wang, X. , Zhang, L. , Chen, S. , Piecychna, M. , Allore, H. , Bockenstedt, L. , Malawista, S. , Bucala, R. , Shaw, A. C. , Fikrig, E. , & Montgomery, R. R. (2012). Age‐associated elevation in TLR5 leads to increased inflammatory responses in the elderly. Aging Cell, 11, 104–110. 10.1111/j.1474-9726.2011.00759.x 22023165 PMC3257374

[acel13316-bib-0136] Quinn, K. M. , Fox, A. , Harland, K. L. , Russ, B. E. , Li, J. , Nguyen, T. H. O. , Loh, L. , Olshanksy, M. , Naeem, H. , Tsyganov, K. , Wiede, F. , Webster, R. , Blyth, C. , Sng, X. Y. X. , Tiganis, T. , Powell, D. , Doherty, P. C. , Turner, S. J. , Kedzierska, K. , & La Gruta, N. L. (2018). Age‐related decline in primary CD8(+) T cell responses is associated with the development of senescence in virtual memory CD8(+) T cells. Cell Reports, 23, 3512–3524. 10.1016/j.celrep.2018.05.057 29924995

[acel13316-bib-0137] Ray, D. , & Yung, R. (2018). Immune senescence, epigenetics and autoimmunity. Clinical Immunology, 196, 59–63. 10.1016/j.clim.2018.04.002 29654845 PMC6548177

[acel13316-bib-0138] Robinson, K. M. , Janes, M. S. , Pehar, M. , Monette, J. S. , Ross, M. F. , Hagen, T. M. , Murphy, M. P. , & Beckman, J. S. (2006). Selective fluorescent imaging of superoxide in vivo using ethidium‐based probes. Proceedings of the National Academy of Sciences of the United States of America, 103, 15038–15043. 10.1073/pnas.0601945103 17015830 PMC1586181

[acel13316-bib-0139] Rubinsztein, D. C. , Marino, G. , & Kroemer, G. (2011). Autophagy and aging. Cell, 146, 682–695. 10.1016/j.cell.2011.07.030 21884931

[acel13316-bib-0140] Rubtsov, A. V. , Rubtsova, K. , Fischer, A. , Meehan, R. T. , Gillis, J. Z. , Kappler, J. W. , & Marrack, P. (2011). Toll‐like receptor 7 (TLR7)‐driven accumulation of a novel CD11c(+) B‐cell population is important for the development of autoimmunity. Blood, 118, 1305–1315. 10.1182/blood-2011-01-331462 21543762 PMC3152497

[acel13316-bib-0141] Sagiv, A. , Burton, D. G. A. , Moshayev, Z. , Vadai, E. , Wensveen, F. , Ben‐Dor, S. , Golani, O. , Polic, B. , & Krizhanovsky, V. (2016). NKG2D ligands mediate immunosurveillance of senescent cells. Aging (Albany NY), 8, 328–344. 10.18632/aging.100897 26878797 PMC4789586

[acel13316-bib-0142] Salam, N. et al (2013). T cell ageing: effects of age on development, survival & function. Indian Journal of Medical Research, 138, 595–608. PMC3928693 24434315

[acel13316-bib-1003] Sampson, M. J. , Winterbone, M. S. , Hughes, J. C. , Dozio, N. , & Hughes, D. A. (2006). Monocyte telomere shortening and oxidative DNA damage in type 2 diabetes. Diabetes Care, 29(2), 283–289. 16443874 10.2337/diacare.29.02.06.dc05-1715

[acel13316-bib-0143] Sandalio, L. M. , Rodriguez‐Serrano, M. , Romero‐Puertas, M. C. , & del Rio, L. A. (2013). Role of peroxisomes as a source of reactive oxygen species (ROS) signaling molecules. SubCellular Biochemistry, 69, 231–255. 10.1007/978-94-007-6889-5_13 23821152

[acel13316-bib-0144] Sanderson, S. L. , & Simon, A. K. (2017). In aged primary T cells, mitochondrial stress contributes to telomere attrition measured by a novel imaging flow cytometry assay. Aging Cell, 16, 1234–1243. 10.1111/acel.12640 28834142 PMC5676074

[acel13316-bib-0145] Schieber, M. , & Chandel, N. S. (2014). ROS function in redox signaling and oxidative stress. Current Biology, 24, R453–462. 10.1016/j.cub.2014.03.034 24845678 PMC4055301

[acel13316-bib-0146] Schipper‐Krom, S. , Sanz, A. S. , van Bodegraven, E. J. , Speijer, D. , Florea, B. I. , Ovaa, H. , & Reits, E. A. (2019). Visualizing proteasome activity and intracellular localization using fluorescent proteins and activity‐based probes. Frontiers in Molecular Biosciences, 6, 56 10.3389/fmolb.2019.00056 31482094 PMC6710370

[acel13316-bib-0147] Sebastian, C. , Herrero, C. , Serra, M. , Lloberas, J. , Blasco, M. A. , & Celada, A. (2009). Telomere shortening and oxidative stress in aged macrophages results in impaired STAT5a phosphorylation. The Journal of Immunology, 183, 2356–2364. 10.4049/jimmunol.0901131 19605693

[acel13316-bib-0148] Sedelnikova, O. A. , Rogakou, E. P. , Panyutin, I. G. , & Bonner, W. M. (2002). Quantitative detection of (125)IdU‐induced DNA double‐strand breaks with gamma‐H2AX antibody. Radiation Research, 158, 486–492.https://doi.org/10.1667/0033‐7587(2002)158[0486:qdoiid]2.0.co;2 12236816 10.1667/0033-7587(2002)158[0486:qdoiid]2.0.co;2

[acel13316-bib-0149] Seidler, S. , Zimmermann, H. W. , Bartneck, M. , Trautwein, C. , & Tacke, F. (2010). Age‐dependent alterations of monocyte subsets and monocyte‐related chemokine pathways in healthy adults. BMC Immunology, 11, 30 10.1186/1471-2172-11-30 20565954 PMC2910032

[acel13316-bib-0150] Shay, J. W. , & Wright, W. E. (2019). Telomeres and telomerase: Three decades of progress. Nature Reviews Genetics, 20, 299–309. 10.1038/s41576-019-0099-1 30760854

[acel13316-bib-0151] Shimatani, K. , Nakashima, Y. , Hattori, M. , Hamazaki, Y. , & Minato, N. (2009). PD‐1+ memory phenotype CD4+ T cells expressing C/EBPalpha underlie T cell immunodepression in senescence and leukemia. Proceedings of the National Academy of Sciences of the United States of America, 106, 15807–15812. 10.1073/pnas.0908805106. 19805226 PMC2739871

[acel13316-bib-0152] Simon, A. K. , Hollander, G. A. , & McMichael, A. (2015). Evolution of the immune system in humans from infancy to old age. Proceedings of the Royal Society B: Biological Sciences, 282, 20143085 10.1098/rspb.2014.3085 PMC4707740 26702035

[acel13316-bib-1006] Signer, R. A. J. , Magee, J. A. , Salic, A. , Morrison, S. J. (2014). Haematopoietic stem cells require a highly regulated protein synthesis rate. Nature, 509(7498), 49–54. 10.1038/nature13035 24670665 PMC4015626

[acel13316-bib-0153] Singh Kushwaha, S. , Patro, N. , & Kumar Patro, I. (2018). A sequential study of age‐related lipofuscin accumulation in hippocampus and striate cortex of rats. Annals of Neurosciences, 25, 223–233. 10.1159/000490908 31000961 PMC6470335

[acel13316-bib-0154] Stein, G. H. , Drullinger, L. F. , Soulard, A. , & Dulic, V. (1999). Differential roles for cyclin‐dependent kinase inhibitors p21 and p16 in the mechanisms of senescence and differentiation in human fibroblasts. Molecular and Cellular Biology, 19, 2109–2117. 10.1128/mcb.19.3.2109 10022898 PMC84004

[acel13316-bib-0155] Stranks, A. J. , Hansen, A. L. , Panse, I. , Mortensen, M. , Ferguson, D. J. P. , Puleston, D. J. , Shenderov, K. , Watson, A. S. , Veldhoen, M. , Phadwal, K. , Cerundolo, V. , & Simon, A. K. (2015). Autophagy controls acquisition of aging features in macrophages. Journal of Innate Immunity, 7, 375–391. 10.1159/000370112 25764971 PMC4386145

[acel13316-bib-0156] Sun, N. , Youle, R. J. , & Finkel, T. (2016). The mitochondrial basis of aging. Molecular Cell, 61, 654–666. 10.1016/j.molcel.2016.01.028 26942670 PMC4779179

[acel13316-bib-0157] Tedone, E. , Huang, E. , O’Hara, R. , Batten, K. , Ludlow, A. T. , Lai, T.‐P. , Arosio, B. , Mari, D. , Wright, W. E. , & Shay, J. W. (2019). Telomere length and telomerase activity in T cells are biomarkers of high‐performing centenarians. Aging Cell, 18, e12859 10.1111/acel.12859 30488553 PMC6351827

[acel13316-bib-0158] Terman, A. , & Brunk, U. T. (2004). Lipofuscin. International Journal of Biochemistry & Cell Biology, 36, 1400–1404. 10.1016/j.biocel.2003.08.009 15147719

[acel13316-bib-0159] Terman, A. , Dalen, H. , & Brunk, U. T. (1999). Ceroid/lipofuscin‐loaded human fibroblasts show decreased survival time and diminished autophagocytosis during amino acid starvation. Experimental Gerontology, 34, 943–957. 10.1016/s0531-5565(99)00070-4 10673148

[acel13316-bib-0160] Thevarajan, I. , Nguyen, T. H. O. , Koutsakos, M. , Druce, J. , Caly, L. , van de Sandt, C. E. , Jia, X. , Nicholson, S. , Catton, M. , Cowie, B. , Tong, S. Y. C. , Lewin, S. R. , & Kedzierska, K. (2020). Breadth of concomitant immune responses prior to patient recovery: A case report of non‐severe COVID‐19. Nature Medicine, 26, 453–455. 10.1038/s41591-020-0819-2 PMC7095036 32284614

[acel13316-bib-0161] Uhl, B. , Vadlau, Y. , Zuchtriegel, G. , Nekolla, K. , Sharaf, K. , Gaertner, F. , Massberg, S. , Krombach, F. , & Reichel, C. A. (2016). Aged neutrophils contribute to the first line of defense in the acute inflammatory response. Blood, 128, 2327–2337. 10.1182/blood-2016-05-718999 27609642 PMC5122310

[acel13316-bib-0162] Valdor, R. , Mocholi, E. , Botbol, Y. , Guerrero‐Ros, I. , Chandra, D. , Koga, H. , Gravekamp, C. , Cuervo, A. M. , & Macian, F. (2014). Chaperone‐mediated autophagy regulates T cell responses through targeted degradation of negative regulators of T cell activation. Nature Immunology, 15, 1046–1054. 10.1038/ni.3003 25263126 PMC4208273

[acel13316-bib-0163] Verma, K. , Ogonek, J. , Varanasi, P. R. , Luther, S. , Bünting, I. , Thomay, K. , Behrens, Y. L. , Mischak‐Weissinger, E. , & Hambach, L. (2017). Human CD8+ CD57‐ TEMRA cells: Too young to be called "old". PLoS One, 12, e0177405 10.1371/journal.pone.0177405 28481945 PMC5421808

[acel13316-bib-0164] Vida, C. , de Toda, I. M. , Cruces, J. , Garrido, A. , Gonzalez‐Sanchez, M. , & De la Fuente, M. (2017). Role of macrophages in age‐related oxidative stress and lipofuscin accumulation in mice. Redox Biology, 12, 423–437. 10.1016/j.redox.2017.03.005 28319893 PMC5357673

[acel13316-bib-0165] von Zglinicki, T. , Nilsson, E. , Docke, W. D. , & Brunk, U. T. (1995). Lipofuscin accumulation and ageing of fibroblasts. Gerontology, 41(Suppl 2), 95–108. 10.1159/000213728 8821324

[acel13316-bib-0166] Walter, P. , & Ron, D. (2011). The unfolded protein response: From stress pathway to homeostatic regulation. Science, 334, 1081–1086. 10.1126/science.1209038 22116877

[acel13316-bib-0167] Weinberger, B. (2018). Vaccines for the elderly: Current use and future challenges. Immun Ageing, 15, 3 10.1186/s12979-017-0107-2 29387135 PMC5778733

[acel13316-bib-0168] Wenisch, C. , Patruta, S. , Daxbock, F. , Krause, R. , & Horl, W. (2000). Effect of age on human neutrophil function. Journal of Leukocyte Biology, 67, 40–45. 10.1002/jlb.67.1.40. 10647996

[acel13316-bib-0169] Xu, W. et al (2019). Mapping of gamma/delta T cells reveals Vdelta2+ T cells resistance to senescence. EBioMedicine, 39, 44–58. 10.1016/j.ebiom.2018.11.053 30528453 PMC6354624

[acel13316-bib-0170] Xu, W. , & Larbi, A. (2017). Markers of T cell senescence in humans. International Journal of Molecular Sciences, 18, 10.3390/ijms18081742 PMC5578132 28796199

[acel13316-bib-0171] Yarbro, J. R. , Emmons, R. S. , & Pence, B. D. (2020). Macrophage immunometabolism and inflammaging: Roles of mitochondrial dysfunction, cellular senescence, CD38, and NAD. Immunometabolism, 2, e200026 10.20900/immunometab20200026 32774895 PMC7409778

[acel13316-bib-0172] Ye, J. , Rawson, R. B. , Komuro, R. , Chen, X. , Davé, U. P. , Prywes, R. , Brown, M. S. , & Goldstein, J. L. (2000). ER stress induces cleavage of membrane‐bound ATF6 by the same proteases that process SREBPs. Molecular Cell, 6, 1355–1364. 10.1016/s1097-2765(00)00133-7 11163209

[acel13316-bib-0173] Yue, X. , Zhao, Y. , Xu, Y. , Zheng, M. , Feng, Z. , & Hu, W. (2017). Mutant p53 in cancer: Accumulation, gain‐of‐function, and therapy. Journal of Molecular Biology, 429, 1595–1606. 10.1016/j.jmb.2017.03.030 28390900 PMC5663274

[acel13316-bib-0174] Zhang, H. , Alsaleh, G. , Feltham, J. , Sun, Y. , Napolitano, G. , Riffelmacher, T. , Charles, P. , Frau, L. , Hublitz, P. , Yu, Z. , Mohammed, S. , Ballabio, A. , Balabanov, S. , Mellor, J. , & Simon, A. K. (2019). Polyamines control eIF5A hypusination, TFEB translation, and autophagy to reverse B cell senescence. Molecular Cell, 76(1), 110–125.e9. 10.1016/j.molcel.2019.08.005 31474573 PMC6863385

[acel13316-bib-0175] Zhang, J. , Zeng, H. , Gu, J. , Li, H. , Zheng, L. , & Zou, Q. (2020). Progress and prospects on vaccine development against SARS‐CoV‐2. Vaccines (Basel), 8, 153 10.3390/vaccines8020153 PMC7349596 32235387

[acel13316-bib-0176] Zhang, Y. , Dai, M. , & Yuan, Z. (2018). Methods for the detection of reactive oxygen species. Analytical Methods, 10, 4625–4638.

[acel13316-bib-0177] Zhu, H. , Bannenberg, G. L. , Moldeus, P. , & Shertzer, H. G. (1994). Oxidation pathways for the intracellular probe 2’,7'‐dichlorofluorescein. Archives of Toxicology, 68, 582–587. 10.1007/s002040050118 7998826

[acel13316-bib-0178] Zhu, Y. , Tchkonia, T. , Pirtskhalava, T. , Gower, A. C. , Ding, H. , Giorgadze, N. , Palmer, A. K. , Ikeno, Y. , Hubbard, G. B. , Lenburg, M. , O'Hara, S. P. , LaRusso, N. F. , Miller, J. D. , Roos, C. M. , Verzosa, G. C. , LeBrasseur, N. K. , Wren, J. D. , Farr, J. N. , Khosla, S. , … Kirkland, J. L. (2015). The Achilles’ heel of senescent cells: from transcriptome to senolytic drugs. Aging Cell, 14(4), 644–658. 10.1111/acel.12344 25754370 PMC4531078

[acel13316-bib-0179] Zielonka, J. , Srinivasan, S. , Hardy, M. , Ouari, O. , Lopez, M. , Vasquez‐Vivar, J. , Avadhani, N. G. , & Kalyanaraman, B. (2008). Cytochrome c‐mediated oxidation of hydroethidine and mito‐hydroethidine in mitochondria: Identification of homo‐ and heterodimers. Free Radical Biology and Medicine, 44, 835–846. 10.1016/j.freeradbiomed.2007.11.013 18155177 PMC2692199

